# Risk factors for Gram-negative bacterial bloodstream infections: a systematic review and meta-analysis with subgroup analyses for carbapenem-resistant organisms and *Acinetobacter baumannii*

**DOI:** 10.1186/s12879-026-14108-6

**Published:** 2026-08-08

**Authors:** Wang Zhang, Mengke Ye, Wenzeng Xu, Xiaoting Hua, Hua Zhou, Yunsong Yu

**Affiliations:** 1 Department of Respiratory and Critical Care Medicine, Sir Run Run Shaw Hospital, Zhejiang University School of Medicine, Hangzhou, China; 2 Department of Infectious Diseases, Sir Run Run Shaw Hospital, Zhejiang University School of Medicine, Hangzhou, China; 3 Department of Critical Care Medicine, Sir Run Run Shaw Hospital, Zhejiang University School of Medicine, Hangzhou, China; 4 Key Laboratory of Microbial Technology and Bioinformatics of Zhejiang Province, Hangzhou, China; 5 Department of Respiratory and Critical Care Medicine, The Second Affiliated Hospital, Zhejiang University School of Medicine, Hangzhou, China

**Keywords:** Gram-negative bacteria, Carbapenem-resistant, *Acinetobacter baumannii*, Bloodstream infection, Risk factors, Meta-analysis

## Abstract

**Background:**

Gram-negative bacterial (GNB) bloodstream infections (BSIs), particularly those caused by carbapenem-resistant GNB (CR-GNB) and *Acinetobacter baumannii* (AB), are associated with substantial morbidity and mortality. This study aimed to provide, to our knowledge, the first comprehensive meta-analysis integrating overall GNB-BSI risk factors with both pathogen-specific and resistance-defined subgroup analyses.

**Methods:**

A systematic literature search was conducted across PubMed, Web of Science, Embase, the Cochrane Library, and four major Chinese databases from inception to August 2025. We included observational studies evaluating risk factors for GNB-related BSIs in patients with GNB infections. Two reviewers independently performed study screening, data extraction, and quality assessment. Methodological quality was evaluated using the Newcastle–Ottawa Scale and the Agency for Healthcare Research and Quality tool. Meta-analyses were performed using R software. Pooled odds ratios (ORs) or standardized mean differences (SMDs) with 95% confidence intervals (CIs) were estimated using the Mantel-Haenszel method, applying either fixed-effects or random-effects models based on the degree of heterogeneity. Furthermore, pre-specified subgroup analyses were conducted to explore potential sources of heterogeneity.

**Results:**

Seventy-five studies (76 articles), encompassing 26,377 patients with GNB infections, were included. The pooled incidence of BSI was 17.3%. Of the 29 evaluated risk factors, 17 were significantly associated with GNB-BSI in the overall population. Procalcitonin (SMD, 0.64; 95%CI, 0.39–0.88), C-reactive protein (SMD, 0.63; 95%CI, 0.43–0.83), carbapenem use (OR, 2.49; 95%CI, 1.92–3.23), mechanical ventilation (OR, 2.51; 95%CI, 1.74–3.61), central venous catheterization (OR, 2.67; 95%CI, 2.27–3.15), immunosuppressive status (OR, 2.20; 95%CI, 1.50–3.22), and aminoglycoside use (OR, 1.68; 95%CI, 1.31–2.15) emerged as the most consistent risk predictors of GNB-BSI. Subgroup analyses indicated that CR-GNB–BSI was specifically associated with hypoalbuminemia and indwelling urinary catheter use, whereas continuous renal replacement therapy was uniquely associated with AB-BSI.

**Conclusions:**

As the first meta-analysis to integrate pathogen-specific and resistance-defined subgroups, this study delineates distinct risk profiles for GNB-BSI. While biomarkers of inflammation, prior carbapenem exposure, and invasive procedures represent broadly shared predictors, CR-GNB–BSI and AB-BSI exhibit additional, subtype-specific risk factors. These findings assist clinicians in the early identification of BSIs among patients with GNB infections.

**Registration number:**

CRD420251117325.

**Clinical trial number:**

Not applicable.

**Supplementary Information:**

The online version contains supplementary material available at https://doi.org/10.1186/s12879-026-14108-6.

## Introduction

Bloodstream infections (BSIs) caused by Gram-negative bacteria (GNB) constitute a major global public health concern and are associated with high mortality rates [1, 2]. Among GNB, *Acinetobacter baumannii* (AB), *Enterobacter* species (Ent.), *Klebsiella pneumoniae* (KP), and *Pseudomonas aeruginosa* (PA) represent the most frequently implicated pathogens. Of particular concern is the rapid emergence and dissemination of carbapenem-resistant GNB (CR-GNB), which severely undermines the effectiveness of last-line antimicrobial therapies. Global surveillance studies indicate that CR-GNB account for a substantial and increasing proportion of BSI cases [3, 4]. This threat is especially pronounced for AB. Data from the China Antimicrobial Surveillance Network (CHINET) spanning 2020 to 2024 demonstrate persistently high rates of carbapenem-resistant AB (CRAB), increasing from 53.7% to 55.2%, markedly exceeding the prevalence observed for carbapenem-resistant KP (CRKP; approximately 10%) and carbapenem-resistant Ent. (CRE) species (< 1%) [5]. 

AB warrants focused investigation owing to its diverse resistance mechanisms and strong environmental adaptability. The organism readily colonizes hospital environments and disproportionately affects vulnerable populations, including intensive care unit (ICU) patients, individuals with hematological malignancies, and those undergoing invasive interventions such as CRRT or prolonged catheterization [3, 6]. In the context of CRAB, progression from colonization to bloodstream infection is facilitated by well-recognized factors, including prior colonization and mechanical ventilation [7]. Clinical outcomes associated with AB-BSI are particularly poor; recent cohort studies report mortality rates as high as 74%, substantially exceeding those observed with other GNB infections [8]. This excess mortality is strongly associated with treatment failure and poor prognosis due to its extensive resistance spectrum [9, 10]. Furthermore, the pronounced biofilm-forming capacity of AB complicates catheter management and renders eradication strategies challenging. Collectively, these pathogen-specific characteristics highlight the necessity of identifying precise and differentiated risk factors for AB-BSI.

Despite an expanding body of literature addressing GNB-BSI, comprehensive evaluations of risk factors across multiple GNB species and resistance phenotypes remain limited. Existing meta-analyses often concentrate on single pathogens or narrowly defined patient populations, thereby failing to capture the broader epidemiological landscape [11, 12]. This limitation constrains the development of precise prevention and management strategies. Accordingly, the present meta-analysis aims to systematically evaluate overall risk factors for GNB-BSI, delineate pathogen-specific risk profiles for AB, KP, Ent., PA, and identify distinct predictors associated with carbapenem-resistant phenotypes, including CR-GNB and CRAB. By differentiating shared and subtype-specific determinants, this study seeks to provide a robust evidence base for tailored prevention strategies and optimized antimicrobial stewardship (AMS).

## Methods

### Search strategy

The protocol for this systematic review and meta-analysis was prospectively registered with the International Prospective Register of Systematic Reviews (PROSPERO; registration number CRD420251117325). A comprehensive search was conducted in PubMed, Web of Science, Embase, the Cochrane Library, China National Knowledge Infrastructure, WanFang Data, VIP Database, and the China Biology Medicine Database to identify studies examining risk factors for GNB-associated BSI. The final search was completed on August 4, 2025, without restrictions on publication date. Studies published in English, Japanese, or Chinese were eligible. Detailed search strategies are provided in Supplementary Table 6.

Two authors (Wang Zhang and Mengke Ye) independently screened all retrieved records and evaluated full-text articles for eligibility. When key information was unclear or missing, corresponding authors were contacted to obtain clarification.

### Inclusion and exclusion criteria

Eligible studies were observational analytical investigations assessing risk factors, predictors, or exposure variables for BSI caused by GNB, specifically AB, Ent., KP, and PA. The study population comprised hospitalized patients with exposure to, colonization by, or infection with these organisms, and each study was required to include a comparator group of patients without BSI. To ensure suitability for quantitative synthesis, studies had to report odds ratio (OR) with 95% confidence interval (CI) for categorical variables or provide sufficient data for their calculation. For continuous variables, mean and standard deviation (SD) were required or had to be derivable from the reported data. Duplicate publications, in vitro or animal studies, case reports, case series, and review articles were excluded.

### Assessment of study quality

The methodological quality of included cohort and case-control studies was assessed using the Newcastle–Ottawa Scale (NOS), with scores ranging from 0 to 9. Cross-sectional studies were evaluated using the Agency for Healthcare Research and Quality (AHRQ) checklist, which yields scores from 0 to 11. Quality assessments were conducted independently by Wang Zhang and Mengke Ye, with discrepancies resolved through consultation with a third author (Wenzeng Xu).

### Data extraction

Two investigators (Wang Zhang and Mengke Ye) independently extracted data from all eligible studies. Extracted information included study characteristics, including the first author, year of publication, country, study period, and design; population characteristics, such as pathogen type and sample size; and outcome data, including frequencies, ORs with 95% CIs for categorical variables, along with descriptive statistics for continuous variables (mean, SD, median, interquartile range, and minimum and maximum values). For categorical variables, ORs were extracted according to a predefined hierarchical strategy to minimize selection bias and maximize data availability: adjusted ORs from multivariable analyses were preferentially used; if these were unavailable, unadjusted ORs from univariable analyses were extracted; if neither was reported, ORs were calculated from the available 2 × 2 table data.

### Data analysis and statistical methods

All statistical analyses were performed using R software (version 4.5.1). Between-study heterogeneity was assessed using the I² statistic, with values exceeding 50% indicating substantial heterogeneity. For dichotomous outcomes, pooled ORs with 95% CIs were calculated, and statistical significance was inferred when the CI did not include 1. For continuous variables, the standardized mean difference (SMD) and its 95% CI were computed, with statistical significance defined by exclusion of 0 from the CI. A fixed-effects model was applied for the meta-analysis, whereas a random-effects model was used when significant heterogeneity (I² > 50%) was present. Subgroup analyses were conducted to explore potential sources of heterogeneity, and differences between subgroups were evaluated using the test for interaction. Publication bias was assessed using Egger’s test. Statistical significance was defined as *p* < 0.05.

## Results

### Study characteristics

**Fig. 1:**
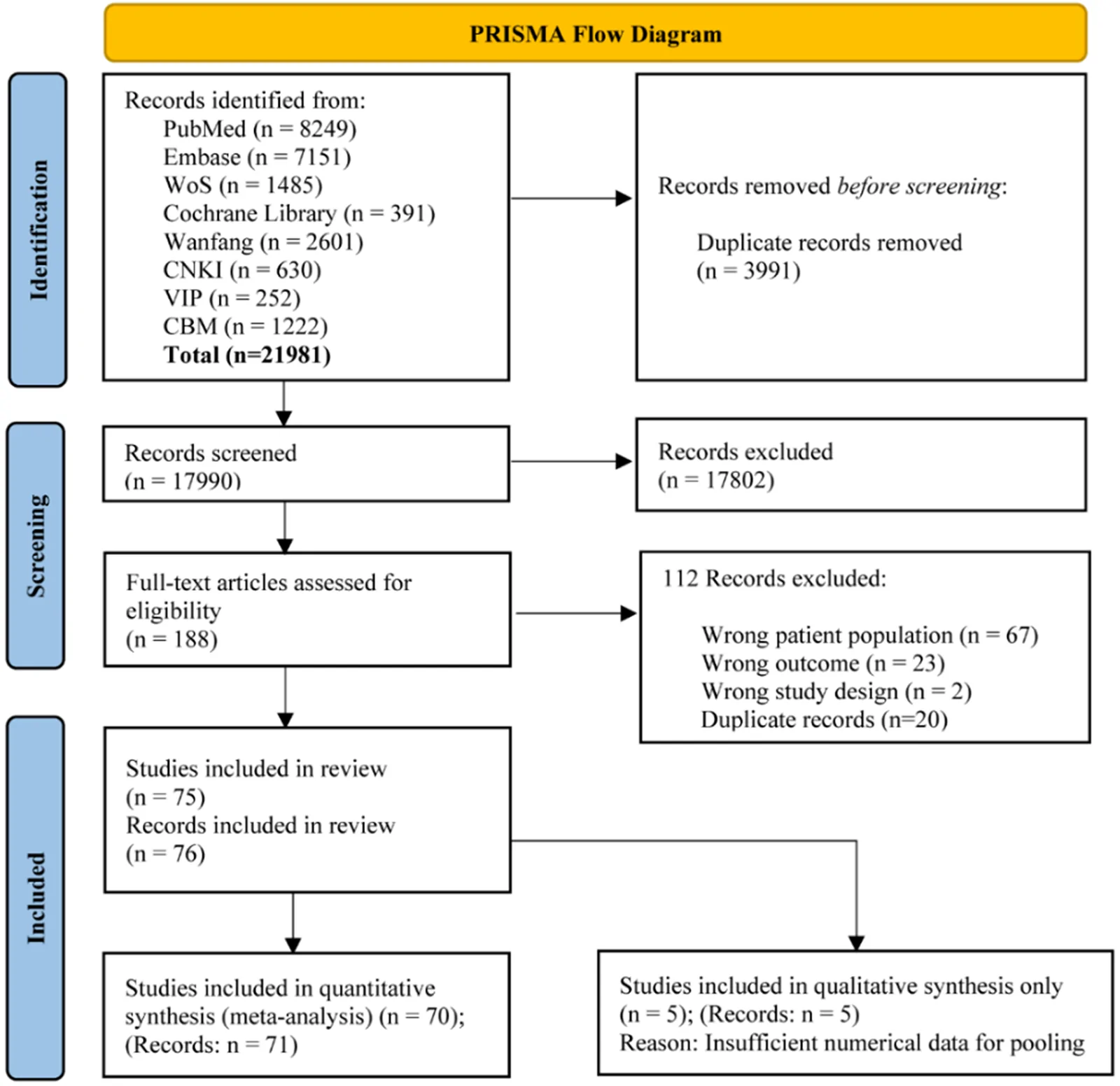
Study flow chart. Note: A total of 75 studies were included in the systematic review. Of these, 70 studies (71 papers) were included in the meta-analysis, while 5 studies (5 papers) were synthesized qualitatively because they did not provide sufficient numerical data for quantitative pooling

A total of 21,981 records were identified through the initial search of eight databases. After duplicate records were removed, 188 articles were retained for full-text review following title and abstract screening. Of these, 112 articles were excluded after full-text assessment for not meeting the eligibility criteria. Ultimately, 75 studies, reported across 76 papers, were included in the final analysis (Fig. 1).

The main characteristics of the included studies are summarized in Table 1. The 75 studies were published between 1992 and 2025 and comprised 39 cohort studies (reported in 40 papers), 34 case-control studies, and 2 cross-sectional studies. The studies were conducted across four continents, with a marked predominance of research from Asia (*n* = 55). Within Asia, most studies were conducted in China (*n* = 43), followed by South Korea (*n* = 6), Israel (*n* = 4), Thailand (*n* = 1), and Saudi Arabia (*n* = 1). This was followed by Europe, which accounted for 13 studies, including those from Italy (*n* = 5), Greece (*n* = 2), the Netherlands (*n* = 2), Germany (*n* = 1), France (*n* = 1), Finland (*n* = 1), and Spain (*n* = 1). Six studies were conducted in North America, including four from the United States (reported in five papers), one from Canada, and one from Mexico. One study was conducted in South America (Brazil). With respect to the target pathogens, Ent. was the most frequently investigated group (*n* = 26), followed by AB (*n* = 20) and KP (*n* = 19). One cohort study contributed two separate publications that examined different pathogens; consequently, GNB overall were reported in six papers, and PA was reported in five papers.

Methodological quality assessment indicated that 60 of the 74 cohort and case-control studies (81.1%) were rated as high quality according to the NOS, while the remaining 14 were assessed as moderate quality (Supplementary Tables 3 and 4). The two cross-sectional studies were both rated as moderate quality based on the AHRQ criteria (Supplementary Table 5). Importantly, none of the included studies were classified as low quality.

Across all included studies, sample sizes ranged from 22 to 6,993 participants, yielding a total population of 26,377 patients with GNB infections. Among these patients, 4,565 (17.3%) developed BSI. Seventy studies (reported in 71 papers) provided sufficient data to be included in the quantitative meta-analysis, encompassing 23,535 patients and demonstrating an overall pooled BSI incidence of 18.5% (4,346 cases).

In total, 29 potential risk factors for GNB-associated BSI were identified and evaluated. These factors encompassed baseline patient characteristics and comorbidities, laboratory parameters measured at admission or at the onset of infection, and medical interventions or clinical exposures. Baseline variables included demographic characteristics, comorbidity burden as assessed by the Charlson Comorbidity Index (CCI), and major chronic conditions such as chronic obstructive pulmonary disease (COPD), heart failure, immunosuppressive state, diabetes mellitus, hypertension, chronic kidney disease (CKD), and malignancy, with cancer further classified as solid or non-solid (hematological) malignancies. Laboratory indicators included inflammatory and hematologic markers such as C-reactive protein (CRP), white blood cell (WBC), procalcitonin (PCT), neutrophil, albumin (ALB), and platelet (PLT). Medical interventions and exposures encompassed invasive and therapeutic factors, including mechanical ventilation (MV) and its duration, immunosuppressive therapy, continuous renal replacement therapy (CRRT), surgery, invasive devices such as central venous catheters (CVCs) and indwelling urinary catheters (IUCs), and exposure to specific antimicrobial classes, including carbapenems, aminoglycosides, cephalosporins, and fluoroquinolones.

**Table 1:** General characteristics of the studies included

Baseline information									Quality assessment	Outcome
Authors	Year	Country	Study period	Design	GNB species	BSI samples	Non-BSI samples	NOS/AHRQ	Grade	
Thamer A. Alenazi et al [13]	2023	Saudi Arabia	2020.3-2021.9	Cohort	AB	94	66	9	high grade	① ② ④ ⑤ ⑦ ⑧ ⑨ ⑩ ⑱ $\bigcirc\!\!\!\!\!\!{24}$
Anucha Apisarnthanarak et al [14]	2012	Thailand	2004.1.1-2011.7.31	Cohort	PA	34	86	7	high grade	--
Diana Averbuch et al [15]	2022	Israel	2014.1.1-2020.3.1	Cohort	Ent.	28	193	8	high grade	② ⑩ ⑪ ⑫ ⑲ $$\bigcirc\!\!\!\!\!\!{22}$$$$\bigcirc\!\!\!\!\!\!{23}$$$$\bigcirc\!\!\!\!\!\!{26}$$$$\bigcirc\!\!\!\!\!\!{27}$$$$\bigcirc\!\!\!\!\!\!{28}$$$$\bigcirc\!\!\!\!\!\!{29}$$
Evgeni Brotfain et al [16]	2016	Israel	2005.1-2011.6	Cohort	AB	46	83	9	high grade	① ② ④ ⑦ ⑨ ⑩ ⑭
Juan Chen et al [17]	2023	China	2018.1.1-2020.12.31	Cohort	KP	135	274	8	high grade	① ② ③ ④ ⑥ ⑦ ⑨ ⑩ ⑪ ⑬ ⑭ ⑮ ⑯ ⑰ ⑱ ⑳ $$\bigcirc\!\!\!\!\!\!{21}$$$$\bigcirc\!\!\!\!\!\!{24}$$$$\bigcirc\!\!\!\!\!\!{25}$$$$\bigcirc\!\!\!\!\!\!{26}$$$$\bigcirc\!\!\!\!\!\!{27}$$
Francesco Cogliati Dezza et al [7]	2023	Italy	2021.1-2022.9	Cohort	CRAB	57	72	9	high grade	① ② ③ ④ ⑤ ⑥ ⑦ ⑧ ⑨ ⑩ ⑪ ⑫ ⑳ $$\bigcirc\!\!\!\!\!\!{24}$$$$\bigcirc\!\!\!\!\!\!{25}$$$$\bigcirc\!\!\!\!\!\!{26}$$
Leandro Cordero et al [18]	2000	USA	1991.1-1998.12	Cohort	GNB	64	163	6	moderate grade	② $\bigcirc\!\!\!\!\!\!{21}$
Leandro Cordero et al [19]	2000	USA	1991–1998	Cohort	PA	15	33	6	moderate grade	② $$\bigcirc\!\!\!\!\!\!{21}$$$$\bigcirc\!\!\!\!\!\!{26}$$
Patricia Cornejo-Juárez et al [20]	2015	Mexico	2012.11.1-2014.1.31	Cohort	Ent.	36	90	7	high grade	① ② ⑩ ⑫
J.W. Timotëus Deelen et al [21]	2020	Netherlands	2013.6-2015.11	Cohort	GNB	758	1196	7	high grade	① ② ③ ⑥ ⑲ $$\bigcirc\!\!\!\!\!\!{25}$$$$\bigcirc\!\!\!\!\!\!{29}$$
Marco Falcone et al [22]	2021	Italy	2018.12-2019.12	Cohort	CRKP	80	597	8	high grade	① ② ③ ④ ⑦ ⑨ ⑩ ⑪ ⑫ ⑲ $$\bigcirc\!\!\!\!\!\!{22}$$$$\bigcirc\!\!\!\!\!\!{25}$$$$\bigcirc\!\!\!\!\!\!{28}$$$$\bigcirc\!\!\!\!\!\!{29}$$
D. R. Giacobbe et al [23]	2016	Italy	2012.1-2014.3	Cohort	CRKP	37	316	9	high grade	② ⑦ ⑩ ⑪ ⑫ ⑲ $$\bigcirc\!\!\!\!\!\!{23}$$$$\bigcirc\!\!\!\!\!\!{25}$$$$\bigcirc\!\!\!\!\!\!{26}$$$$\bigcirc\!\!\!\!\!\!{27}$$$$\bigcirc\!\!\!\!\!\!{28}$$$$\bigcirc\!\!\!\!\!\!{29}$$
Yihai Gu et al.	2021	China	2009–2018	Case-control	AB	47	124	7	high grade	① ② ⑥ ⑲ $$\bigcirc\!\!\!\!\!\!{25}$$$$\bigcirc\!\!\!\!\!\!{26}$$$$\bigcirc\!\!\!\!\!\!{27}$$
Alicia [24] Herna´ndez-Torres et al [25]	2012	Spain	2007.1-2008.6	Cohort	CRAB	27	50	8	high grade	② ⑤ ⑥ ⑦ ⑧ ⑩ ⑳ $$\bigcirc\!\!\!\!\!\!{25}$$$$\bigcirc\!\!\!\!\!\!{26}$$$$\bigcirc\!\!\!\!\!\!{27}$$
Jessica R. Howard-Anderson et al [26]	2020	USA	2012.1.1-2017.12.31	Cohort	CRE	21	404	9	high grade	① ② $$\bigcirc\!\!\!\!\!\!{26}$$$$\bigcirc\!\!\!\!\!\!{27}$$
Risto Ikäheimo et al [27]	2009	Finland	1989.6-1990.9	Cohort	Ent.	15	34	6	moderate grade	⑬ ⑭
Ji Ye Jung et al [28]	2010	Korea	2008.1-2009.12	Cohort	AB	108	92	9	high grade	① ⑥ ⑦ ⑧ ⑩ ⑪ ⑫ ⑲ ⑳ $$\bigcirc\!\!\!\!\!\!{23}$$$$\bigcirc\!\!\!\!\!\!{24}$$$$\bigcirc\!\!\!\!\!\!{26}$$$$\bigcirc\!\!\!\!\!\!{27}$$$$\bigcirc\!\!\!\!\!\!{28}$$$$\bigcirc\!\!\!\!\!\!{29}$$
Sung-Woon Kang et al [29]	2023	Korea	2018.1-2022.2	Cohort	Ent.	69	1104	9	high grade	① ② ⑦ ⑨ ⑩ ⑪ ⑫
Sun-young Kim et al [30]	2019	Korea	2010.1-2016.12	Cohort	AB	47	394	9	high grade	② ⑥ $\bigcirc\!\!\!\!\!\!{26}$
Konstantina Kontopoulou et al [31]	2019	Greece	2011.6-2014.8	Case-control	CRKP	48	178	6	moderate grade	⑤ ⑥ ⑦ ⑧ ⑲ $$\bigcirc\!\!\!\!\!\!{23}$$$$\bigcirc\!\!\!\!\!\!{25}$$$$\bigcirc\!\!\!\!\!\!{28}$$$$\bigcirc\!\!\!\!\!\!{29}$$
B. J. Liss et al [32]	2012	Germany	2008.9.1-2009.6.22	Cohort	Ent.	6	84	9	high grade	--
Jia Liu et al [33]	2023	China	2017.10.10-2022.7.31	Cohort	CRE	59	362	8	high grade	① ② ⑲ $$\bigcirc\!\!\!\!\!\!{22}$$$$\bigcirc\!\!\!\!\!\!{23}$$$$\bigcirc\!\!\!\!\!\!{29}$$
Jonas Marschall et al [34]	2012	USA	2009.8.1-2010.7.31	Cohort	Ent.	24	132	8	high grade	① ② ③ ⑦ ⑩ $\bigcirc\!\!\!\!\!\!{25}$
Haiming Niu et al [35]	2023	China	2019.1-2021.12	Case-control	CRAB	40	119	7	high grade	① ② ④ ⑤ ⑦ ⑧ ⑨ ⑩ ⑪ ⑬ ⑭ ⑮ ⑯ ⑰ ⑱
Seon Jung Oh et al [36]	2016	Korea	2003.1-2013.11	Case-control	Ent.	34	94	4	moderate grade	① ②
Emmanuel Roilides et al [37]	1992	USA	1986.12-1991.9	Case-control	PA	9	13	6	moderate grade	① ②
Alessandro Russo et al [38]	2021	Italy	2019.1-2021.1	cross-sectional	AB	27	120	7	moderate grade	① ② ③ ④ ⑤ ⑦ ⑨ ⑩ ⑪ ⑫ ⑭ ⑮ $$\bigcirc\!\!\!\!\!\!{24}$$$$\bigcirc\!\!\!\!\!\!{25}$$
Xiangyuan Sun et al [39]	2023	China	2012.1-2020.12	Case-control	CRKP	96	230	7	high grade	① ② ⑦ ⑧ ⑩ ⑲ $$\bigcirc\!\!\!\!\!\!{23}$$$$\bigcirc\!\!\!\!\!\!{26}$$$$\bigcirc\!\!\!\!\!\!{27}$$$$\bigcirc\!\!\!\!\!\!{28}$$$$\bigcirc\!\!\!\!\!\!{29}$$
Elizabeth Temkin et al [40]	2023	Israel	2020.1.1-2022.10.10	Cohort	Ent.	165	6828	9	high grade	① ②
Chin-Chung Tseng et al [41]	2002	China	1997.1-1999.12	Cohort	Ent.	54	27	8	high grade	② ⑥ ⑦
Willize E. van der Starre et al [42]	2015	Netherlands	--	Case-control	Ent.	46	45	6	moderate grade	① ② ④ ⑤ ⑦ ⑧ ⑨ ⑩ $$\bigcirc\!\!\!\!\!\!{22}$$$$\bigcirc\!\!\!\!\!\!{27}$$
A. G. Venier et al [43]	2007	France	2002.10.1-2003.3.1	Case-control	Ent.	24	46	6	moderate grade	① ② ⑥ ⑦ ⑩ $$\bigcirc\!\!\!\!\!\!{26}$$$$\bigcirc\!\!\!\!\!\!{27}$$
Sheng-Huei Wang et al [44]	2024	China	2015–2019	Case-control	CRAB	164	519	8	high grade	① ② ⑤ ⑦ ⑨ ⑩ ⑬ ⑰ ⑳
Su Xu et al [45]	2018	China	2004.1-2013.12	Case-control	AB	102	204	8	high grade	① ② ⑦ ⑩ ⑲ ⑳ $$\bigcirc\!\!\!\!\!\!{25}$$$$\bigcirc\!\!\!\!\!\!{27}$$
Young Kyung Yoon et al [46]	2013	Korea	2008.5-2012.4	Cohort	AB	21	91	9	high grade	① ② ③ ⑩ ⑬ ⑲ ⑳ $$\bigcirc\!\!\!\!\!\!{23}$$$$\bigcirc\!\!\!\!\!\!{25}$$$$\bigcirc\!\!\!\!\!\!{27}$$$$\bigcirc\!\!\!\!\!\!{28}$$$$\bigcirc\!\!\!\!\!\!{29}$$
Hongchen Zhang et al [47]	2023	China	2020.1.1-2022.3.31	Case-control	CRGNB	90	90	8	high grade	① ② ⑦ ⑩ ⑪ ⑲ $$\bigcirc\!\!\!\!\!\!{28}$$$$\bigcirc\!\!\!\!\!\!{29}$$
Li Yan et al [48]	2016	China	2011–2013	Case-control	Ent.	82	82	9	high grade	⑦ $$\bigcirc\!\!\!\!\!\!{25}$$$$\bigcirc\!\!\!\!\!\!{27}$$
Shuhai Shi et al [49]	2023	China	2012.6-2020.10	Cohort	Ent.	84	148	7	high grade	① ② ④ ⑦ ⑧ ⑨ ⑬ ⑭ ⑮ ⑯ ⑱ $\bigcirc\!\!\!\!\!\!{25}$
Liping Huang et al [50]	2022	China	2017.6.1-2019.5.31	Case-control	KP	114	335	8	high grade	② ⑤ ⑦ ⑧ ⑨ ⑩ $$\bigcirc\!\!\!\!\!\!{22}$$$$\bigcirc\!\!\!\!\!\!{25}$$$$\bigcirc\!\!\!\!\!\!{26}$$$$\bigcirc\!\!\!\!\!\!{27}$$
Xiongneng Mou et al [51]	2023	China	2016.1-2022.12	Case-control	CRE	28	64	8	high grade	② ⑦ ⑲
Jingfu Liu et al [52]	2022	China	2013.1-2020.12	Case-control	KP	70	136	7	high grade	① ② ⑦ ⑧ ⑩ ⑬ ⑭ ⑮ ⑯
Miaomiao Wu et al [53]	2022	China	2019.1-2021.12	cross-sectional	CRE	37	161	7	moderate grade	① ② ⑦ ⑧ ⑩ ⑰ ⑲ $$\bigcirc\!\!\!\!\!\!{22}$$$$\bigcirc\!\!\!\!\!\!{26}$$
Rui Wang et al [54]	2024	China	2019.2-2023.12	Case-control	PA	33	84	7	high grade	① ② ⑦ ⑬ ⑭ ⑮
Chunhua Li et al [55]	2014	China	2009–2011	Case-control	PA	11	189	7	high grade	④ ⑦ ⑨
Jing Shi et al [56]	2022	China	2015–2020	Case-control	CRE	172	110	7	high grade	① ⑲ $$\bigcirc\!\!\!\!\!\!{21}$$$$\bigcirc\!\!\!\!\!\!{23}$$$$\bigcirc\!\!\!\!\!\!{29}$$
Weiqun He [57]	2016	China	2011.1-2015.12	Case-control	AB	51	561	8	high grade	① ② ④ ⑤ ⑦ ⑨ $$\bigcirc\!\!\!\!\!\!{22}$$$$\bigcirc\!\!\!\!\!\!{25}$$$$\bigcirc\!\!\!\!\!\!{26}$$
Ying Zhou et al [58]	2025	China	2022.1-2023.11	Case-control	KP	18	82	6	moderate grade	① ② ⑦ ⑧ ⑩ ⑬ ⑭ ⑮ ⑰ ⑲ $$\bigcirc\!\!\!\!\!\!{25}$$$$\bigcirc\!\!\!\!\!\!{27}$$$$\bigcirc\!\!\!\!\!\!{28}$$$$\bigcirc\!\!\!\!\!\!{29}$$
Chunhui Xu et al [59]	2018	China	2016.1-2017.12	Cohort	CRE	13	61	8	high grade	① ②
Han Meng et al [60]	2023	China	2018.1-2021.12	Cohort	CRE	14	123	9	high grade	① ② ⑦ ⑧ ⑩ $$\bigcirc\!\!\!\!\!\!{22}$$$$\bigcirc\!\!\!\!\!\!{25}$$
Jianping Zhao et al [61]	2021	China	2017.1-2019.6	Case-control	CRKP	32	97	6	moderate grade	② ⑦ ⑧ ⑨ ⑩ ⑲ $$\bigcirc\!\!\!\!\!\!{22}$$$$\bigcirc\!\!\!\!\!\!{23}$$$$\bigcirc\!\!\!\!\!\!{25}$$$$\bigcirc\!\!\!\!\!\!{28}$$$$\bigcirc\!\!\!\!\!\!{29}$$
Yalu Ren et al [62]	2023	China	2020.9-2022.6	Cohort	CRE	10	99	6	moderate grade	② ⑩ ⑫
Yuan Zhan et al [63]	2024	China	2020.1-2023.12	Case-control	KP	61	67	7	high grade	① ② ④ ⑦ ⑧ ⑬ ⑭ ⑮ ⑯ ⑰ ⑱ ⑳ $\bigcirc\!\!\!\!\!\!{27}$
Haitao Ren et al [64]	2019	China	2015.1.1-2017.12.31	Case-control	KP	32	59	8	high grade	② $\bigcirc\!\!\!\!\!\!{27}$
Shujuan Chen et al [65]	2024	China	2021.3-2023.3	Cohort	CRE	25	111	9	high grade	① ② ⑦ ⑧ ⑩ ⑰ ⑲ $$\bigcirc\!\!\!\!\!\!{22}$$$$\bigcirc\!\!\!\!\!\!{25}$$$$\bigcirc\!\!\!\!\!\!{26}$$
Guoyao Ding [66]	2023	China	2017.1.1-2021.12.31	Cohort	KP	35	217	9	high grade	② ④ ⑦ ⑧ ⑩ ⑪ ⑫
Fawudan ABUDU [67]	2021	China	2017.1.1-2019.12.31	Case-control	GNB	44	62	7	high grade	② ⑩ ⑲ ⑳ $$\bigcirc\!\!\!\!\!\!{22}$$$$\bigcirc\!\!\!\!\!\!{25}$$$$\bigcirc\!\!\!\!\!\!{26}$$$$\bigcirc\!\!\!\!\!\!{27}$$$$\bigcirc\!\!\!\!\!\!{28}$$
Jihong Li et al [68]	2017	China	2015.1-2016.12	Case-control	AB	52	2059	6	moderate grade	--
Beizhou Lin et al [69]	2014	China	2012.1-2013.1	Case-control	AB	37	53	8	high grade	② ④ ⑦ ⑧ ⑩ ⑬ ⑮ ⑲ ⑳ $$\bigcirc\!\!\!\!\!\!{21}$$$$\bigcirc\!\!\!\!\!\!{24}$$$$\bigcirc\!\!\!\!\!\!{26}$$$$\bigcirc\!\!\!\!\!\!{27}$$$$\bigcirc\!\!\!\!\!\!{28}$$$$\bigcirc\!\!\!\!\!\!{29}$$
Jiaping Lin [70]	2020	China	2017.6.1-2019.6.1	Case-control	KP	114	335	8	high grade	① ② ⑤ ⑦ ⑧ ⑨ ⑩ $$\bigcirc\!\!\!\!\!\!{22}$$$$\bigcirc\!\!\!\!\!\!{25}$$$$\bigcirc\!\!\!\!\!\!{27}$$
Zhen Qi et al [71]	2022	China	2017.6-2021.6	Case-control	CRKP	37	31	6	moderate grade	① ② ③ ⑥ ⑦ ⑧ ⑨ ⑩ ⑭ ⑯ ⑰ ⑱ ⑲ ⑳ $$\bigcirc\!\!\!\!\!\!{24}$$$$\bigcirc\!\!\!\!\!\!{25}$$$$\bigcirc\!\!\!\!\!\!{27}$$
Yuanye Qu [72]	2024	China	2020.10-2022.9	Cohort	CRE	14	78	8	high grade	① ② ⑦ ⑧ ⑩ ⑫ ⑭ ⑲ $$\bigcirc\!\!\!\!\!\!{23}$$$$\bigcirc\!\!\!\!\!\!{28}$$$$\bigcirc\!\!\!\!\!\!{29}$$
Kefan Chen et al [73]	2016	China	2013.1-2015.1	Case-control	KP	12	34	7	high grade	⑦ ⑳
Hongmei Jiang et al [74]	2021	China	2018.6-2019.12	Cohort	CRKP	5	18	7	high grade	① ② ⑩ ⑪ $\bigcirc\!\!\!\!\!\!{24}$
Xinyun Zhu et al [75]	2021	China	2017.1-2019.9	Cohort	CRKP	18	58	8	high grade	① ② ⑬ ⑭ ⑮ ⑯ ⑱ $$\bigcirc\!\!\!\!\!\!{26}$$$$\bigcirc\!\!\!\!\!\!{27}$$
Chiyu Chen et al [76]	2012	China	2008.5.1-2010.11.30	Case-control	GNB	67	124	8	high grade	① ② ③ ④ ⑦ ⑩ ⑬ ⑭
Matthaios Papadimitriou-Olivgeris et al [77]	2017	Greece	2009.11-2011.2	Cohort	KP	24	116	8	high grade	① ② ④ ⑤ ⑦ ⑨ ⑩ ⑲ ⑳ $$\bigcirc\!\!\!\!\!\!{23}$$$$\bigcirc\!\!\!\!\!\!{25}$$$$\bigcirc\!\!\!\!\!\!{28}$$$$\bigcirc\!\!\!\!\!\!{29}$$
HyeonJin Choi et al [78]	2022	Korea	2019.6	Cohort	Ent.	49	587	7	high grade	② ⑦ ⑨ ⑩ ⑪ ⑲ ⑳ $$\bigcirc\!\!\!\!\!\!{22}$$$$\bigcirc\!\!\!\!\!\!{23}$$$$\bigcirc\!\!\!\!\!\!{27}$$$$\bigcirc\!\!\!\!\!\!{28}$$$$\bigcirc\!\!\!\!\!\!{29}$$
Luba Tau et al [79]	2022	Israel	2007.1-2020.6	Case-control	GNB	76	149	8	high grade	① ② ③ ⑥ ⑦ ⑨ ⑩ ⑪ ⑫
A.P. Gray et al [80]	2016	Canada	2012.3-2014.1	Case-control	CRAB	5	23	8	high grade	⑩ ⑫ $\bigcirc\!\!\!\!\!\!{26}$
V. Galfo et al [81]	2025	Italy	2020.6-2023.6	Cohort	CRAB	103	180	8	high grade	--
R Passos et al [82]	2011	Brazil	2007.1-2010.12	Cohort	AB	74	111	8	high grade	⑲
Yunfang Tan et al [83]	2017	China	2012.7-2015.7	Cohort	AB	86	89	8	high grade	① ② ④ ⑦ ⑧ ⑩ ⑬ ⑮ ⑲ ⑳ $$\bigcirc\!\!\!\!\!\!{24}$$$$\bigcirc\!\!\!\!\!\!{25}$$$$\bigcirc\!\!\!\!\!\!{26}$$$$\bigcirc\!\!\!\!\!\!{28}$$$$\bigcirc\!\!\!\!\!\!{29}$$
Lipeng Liu et al [84]	2023	China	2008.1-2020.12	Cohort	CRE	19	40	8	high grade	① ② ⑩ ⑫ ⑲ $$\bigcirc\!\!\!\!\!\!{26}$$$$\bigcirc\!\!\!\!\!\!{28}$$$$\bigcirc\!\!\!\!\!\!{29}$$
Minxue Liu et al [85]	2018	China	2011.3-2016.6	Case-control	Ent.	42	84	7	high grade	① ② ⑩ ⑪ ⑬ ⑭ ⑮ ⑯ ⑲ $$\bigcirc\!\!\!\!\!\!{22}$$$$\bigcirc\!\!\!\!\!\!{23}$$$$\bigcirc\!\!\!\!\!\!{28}$$$$\bigcirc\!\!\!\!\!\!{29}$$
Luxia Wang et al [86]	2018	China	2015.6-2015.12	Case-control	AB	14	28	7	high grade	② ⑮ ⑱ $$\bigcirc\!\!\!\!\!\!{24}$$$$\bigcirc\!\!\!\!\!\!{26}$$
Chenfeng Zhao et al [87]	2025	China	2020.1–2022	Cohort	CRKP	24	214	6	moderate grade	--

The 29 outcome risk factors included in this study and their corresponding numbers are listed below: ①: Age; ②: Gender; ③: CCI; ④: COPD; ⑤: Heart failure; ⑥: Immunosuppressive state; ⑦: Diabetes mellitus; ⑧: Hypertension; ⑨: CKD; ⑩: Cancer; ⑪: Cancer-Solid; ⑫: Cancer-Non-solid/ hematological; ⑬: CRP; ⑭: WBC; ⑮: PCT; ⑯: Neutrophil; ⑰: ALB; ⑱: PLT; ⑲: Carbapenems use; ⑳: MV; $\bigcirc\!\!\!\!\!\!{21}$: MV days; $\bigcirc\!\!\!\!\!\!{22}$: Immunosuppression therapy; $\bigcirc\!\!\!\!\!\!{23}$: Aminoglycosides use; $\bigcirc\!\!\!\!\!\!{24}$: CRRT; $\bigcirc\!\!\!\!\!\!{25}$: Surgery; $\bigcirc\!\!\!\!\!\!{26}$: CVC; $\bigcirc\!\!\!\!\!\!{27}$: IUC; $\bigcirc\!\!\!\!\!\!{28}$: Cephalosporins use; $\bigcirc\!\!\!\!\!\!{29}$: Fluoroquinolones use

### Risk factors for BSI in all-patient cohort

The results of the meta-analysis examining risk factors for BSI in the overall patient cohort are presented in Table 2. Of the 29 candidate risk factors evaluated, 17 were found to be significantly associated with the development of BSIs. Among baseline patient characteristics and comorbidities, significant risk factors included male gender (pooled OR = 1.15, 95% CI: 1.06–1.24, *p* < 0.001, I² = 3.2%), immunosuppressive state (pooled OR = 2.20, 95% CI: 1.50–3.22, *p* < 0.001, I² = 80.3%), CKD (pooled OR = 1.35, 95% CI: 1.02–1.78, *p* = 0.035, I² = 57.2%), and malignancy (pooled OR = 1.28, 95% CI: 1.03–1.60, *p* = 0.027, I² = 61.3%), with the association being particularly pronounced for non-solid cancers (pooled OR = 2.00, 95% CI: 1.25–3.20, *p* = 0.004, I² = 67.0%). Regarding laboratory parameters, CRP (pooled SMD = 0.63, 95% CI: 0.43–0.83, *p* < 0.001, I² = 75.0%) and PCT (pooled SMD = 0.64, 95% CI: 0.39–0.88, *p* < 0.001, I² = 82.3%), as well as ALB (pooled SMD = -0.24, 95% CI: −0.34 to − 0.13, *p* < 0.001, I² = 46.0%) and PLT (pooled SMD = − 0.27, 95% CI: −0.50 to − 0.04, *p* = 0.023, I² = 64.5%) were significantly associated with increased BSI risk. Regarding medical interventions and clinical exposures, carbapenem use (pooled OR = 2.49, 95% CI: 1.92–3.23, *p* < 0.001, I² = 74.4%), MV (pooled OR = 2.51, 95% CI: 1.74–3.61, *p* < 0.001, I² = 56.6%), immunosuppressive therapy (pooled OR = 1.73, 95% CI: 1.23–2.43, *p* = 0.002, I² = 67.1%), aminoglycoside use (pooled OR = 1.68, 95% CI: 1.31–2.15, *p* < 0.001, I² = 44.0%), CRRT (pooled OR = 2.28, 95% CI: 1.27–4.11, *p* = 0.006, I² = 82.2%), surgery (pooled OR = 1.43, 95% CI: 1.02–2.00, *p* = 0.040, I² = 83.4%), CVC (pooled OR = 2.67, 95% CI: 2.27–3.15, *p* < 0.001, I² = 47.8%), and IUC (pooled OR = 1.50, 95% CI: 1.08–2.09, *p* = 0.016, I² = 68.7%) were all significantly associated with an increased risk of BSI. Although substantial heterogeneity was observed for several factors, the direction of effect was largely consistent across studies.

PCT and CRP were analyzed as continuous biomarker variables and are therefore interpreted as diagnostic indicators associated with BSI rather than antecedent exposure-related risk factors. For the cancer variable, additional exploratory analyses of potential publication bias, including funnel plot inspection (Supplementary Figs. 1 and 2), trim-and-fill analysis (Supplementary Fig. 3), and leave-one-out sensitivity analysis (Supplementary Fig. 4), are presented in the Supplementary Materials. Supplementary sensitivity analyses restricted to multivariable-adjusted estimates were also conducted for eight eligible variables and are provided in the Supplementary Materials (Supplementary Figs. 5–12). These analyses were broadly consistent with the primary results, with the exception of gender, for which the association was not consistently retained.

**Table 2:** Meta-analysis of risk factors for the entire GNB patient cohort

Type of factor	No. of studies	No. of patients in studies (non-BSI)	I^2^ (%)	*P* value of Heterogeneity	Pooled OR (95%CI) / SMD [95%CI]	*P* value	*P* value of Egger test
*Age	47	3432 (15761)	79.8	< 0.001	0.05 [-0.05; 0.15]	0.333	0.702
Gender, male	62	3819 (18336)	3.2	0.404	1.15 (1.06; 1.24)	< 0.001	0.159
*CCI	10	1282 (2786)	79.2	< 0.001	0.05 [-0.12; 0.22]	0.557	0.159
COPD	17	981 (2940)	68.9	< 0.001	1.04 (0.72; 1.49)	0.849	0.068
HF	12	806 (2516)	0.0	0.606	0.92 (0.71; 1.20)	0.559	0.012
Immunosuppressive state	12	1418 (2633)	80.3	< 0.001	2.20 (1.50; 3.22)	< 0.001	0.276
DM	45	2579 (8417)	38.7	0.005	1.10 (0.99; 1.22)	0.073	0.465
Hypertension	23	1294 (2915)	10.1	0.323	0.93 (0.81; 1.06)	0.263	0.896
CKD	20	1360 (5547)	57.2	< 0.001	1.35 (1.02; 1.78)	0.035	0.147
Cancer	44	2325 (7683)	61.3	< 0.001	1.28 (1.03; 1.60)	0.027	0.001
Cancer-Solid	15	878 (4032)	66.6	< 0.001	0.80 (0.55; 1.16)	0.237	0.202
Cancer-Non-solid	14	601 (3190)	67.0	< 0.001	2.00 (1.25; 3.20)	0.004	0.004
*CRP	14	805 (1873)	75.0	< 0.001	0.63 [0.43; 0.83]	< 0.001	0.923
*WBC	15	707 (1522)	81.6	< 0.001	0.24 [-0.01; 0.49]	0.062	0.620
*PCT	13	665 (1342)	82.3	< 0.001	0.64 [0.39; 0.88]	< 0.001	0.187
*Neutrophil	8	487 (917)	69.9	0.002	0.19 [-0.03; 0.41]	0.092	0.719
*ALB	8	517 (1364)	46.0	0.073	-0.24 [-0.34; -0.13]	< 0.001	0.325
*PLT	8	483 (791)	64.5	0.006	-0.27 [-0.50; -0.04]	0.023	0.958
Carbapenems use	28	2212 (5549)	74.4	< 0.001	2.49 (1.92; 3.23)	< 0.001	0.344
MV	15	964 (2341)	56.6	0.004	2.51 (1.74; 3.61)	< 0.001	0.187
*MV days	5	423 (633)	91.2	< 0.001	-0.09 [-0.58; 0.39]	0.702	0.250
Immunosuppressive therapy	14	735 (3653)	67.1	< 0.001	1.73 (1.23; 2.43)	0.002	0.428
Aminoglycosides use	13	730 (2534)	44.0	0.045	1.68 (1.31; 2.15)	< 0.001	0.759
CRRT	10	600 (843)	82.2	< 0.001	2.28 (1.27; 4.11)	0.006	0.816
Surgery	25	2088 (5526)	83.4	< 0.001	1.43 (1.02; 2.00)	0.040	0.321
CVC	23	1092 (3749)	47.8	0.006	2.67 (2.27; 3.15)	< 0.001	0.063
IUC	23	1298 (3820)	68.7	< 0.001	1.50 (1.08; 2.09)	0.016	0.064
Cephalosporins use	18	873 (3075)	78.9	< 0.001	1.25 (0.83; 1.87)	0.291	0.276
Fluoroquinolones use	20	1818 (4681)	80.7	< 0.001	1.40 (0.99; 1.97)	0.058	0.840

Note: *Indicates a continuous variable. Results are presented as SMD (95% CI)

### Risk factors for BSI in CR cohort

Table 3 summarizes the results of subgroup meta-analyses comparing carbapenem-resistant (CR) and non-carbapenem-resistant (non-CR) cohorts. Distinct patterns of risk factors were observed between these two groups. Seven factors were consistently and significantly associated with BSI in both CR and non-CR patients, including immunosuppressive state, elevated CRP, elevated PCT, carbapenem use, MV, aminoglycoside use, and the presence of a CVC. In contrast, nine factors were significantly associated with BSI only in the non-CR subgroup, namely male gender, CKD, malignancy, non-solid cancers, WBC, PLT, immunosuppressive therapy, CRRT, and surgery. Conversely, ALB levels and IUC use were identified as significant risk factors exclusively in the CR subgroup. In addition, a focused analysis of the CRAB subgroup identified age, non-solid malignancies, elevated CRP, and MV as significant risk factors for BSI. Detailed results of this analysis are provided in Supplementary Table 1.

**Table 3:** Subgroup meta-analysis of risk factors for CR-GNB- and non-CR-GNB-BSI

Risk factors	CR bacteria			Non-CR bacteria		
	Number of studies	Pooled OR (95%CI) / SMD [95%CI]	*P* value	Number of studies	Pooled OR (95%CI) / SMD [95%CI]	*P* value
*Age	18	-0.04 [-0.22; 0.14]	0.645	29	0.10 [-0.02; 0.23]	0.102
Gender, male	22	1.14 (0.99; 1.33)	0.077	40	1.15 (1.05; 1.25)	0.002
*CCI	3	0.21 [-0.36; 0.77]	0.475	7	0.03 [-0.15; 0.20]	0.774
COPD	3	0.81 (0.51; 1.29)	0.376	14	1.08 (0.69; 1.67)	0.743
Heart failure	5	0.98 (0.70; 1.38)	0.905	7	0.85 (0.56; 1.28)	0.439
Immunosuppressive state	4	1.66 (1.15; 2.39)	0.007	8	2.55 (1.49; 4.37)	0.001
Diabetes mellitus	16	1.09 (0.91; 1.30)	0.367	29	1.10 (0.97; 1.25)	0.120
Hypertension	11	0.90 (0.71; 1.14)	0.398	12	0.94 (0.79; 1.11)	0.440
CKD	6	1.15 (0.51; 2.62)	0.734	14	1.40 (1.14; 1.73)	0.002
Cancer	18	1.24 (0.99; 1.55)	0.056	26	1.43 (1.06; 1.93)	0.019
Cancer-Solid	6	0.78 (0.46; 1.35)	0.379	9	0.80 (0.46; 1.39)	0.433
Cancer-Non-solid	7	1.34 (0.84; 2.13)	0.216	7	3.04 (1.80; 5.13)	< 0.001
*CRP	3	0.79 [0.64; 0.95]	< 0.001	12	0.53 [0.25; 0.81]	< 0.001
*WBC	4	0.06 [-0.44; 0.55]	0.824	11	0.30 [0.00; 0.60]	0.047
*PCT	2	0.61 [0.31; 0.91]	< 0.001	11	0.63 [0.34; 0.92]	< 0.001
*Neutrophil	3	0.22 [-0.18; 0.61]	0.287	5	0.17 [-0.12; 0.47]	0.238
*ALB	5	-0.19 [-0.32; -0.06]	0.005	3	-0.36 [-0.85; 0.12]	0.140
*PLT	3	-0.34 [-1.15; 0.46]	0.405	5	-0.27 [-0.43; -0.11]	< 0.001
Carbapenems use	14	2.09 (1.45; 3.00)	< 0.001	14	2.96 (2.04; 4.31)	< 0.001
MV	4	2.27 (1.26; 4.11)	0.007	11	2.68 (1.63; 4.40)	< 0.001
*MV days	--	--	--	4	-0.28 [-0.67; 0.11]	0.160
Immunosuppressive therapy	6	1.79 (0.99; 3.21)	0.052	8	1.71 (1.33; 2.21)	< 0.001
Aminoglycosides use	7	1.40 (1.01; 1.93)	0.042	6	2.16 (1.48; 3.17)	< 0.001
CRRT	3	3.54 (0.65; 19.14)	0.143	7	2.01 (1.09; 3.71)	0.026
Surgery	9	1.39 (0.66; 2.96)	0.388	16	1.49 (1.00; 2.22)	0.047
CVC	10	2.78 (1.65; 4.68)	< 0.001	13	2.91 (2.32; 3.64)	< 0.001
IUC	6	2.46 (1.08; 5.61)	0.033	17	1.29 (0.92; 1.80)	0.135
Cephalosporins use/exposure	8	0.94 (0.48; 1.82)	0.846	10	1.54 (0.92; 2.56)	0.102
Fluoroquinolones use	10	1.45 (0.84; 2.51)	0.184	10	1.33 (0.84; 2.10)	0.218

Note: *Indicates a continuous variable. Results are presented as SMD (95% CI)

### Risk factors for BSI in AB cohort

**Fig. 2:**
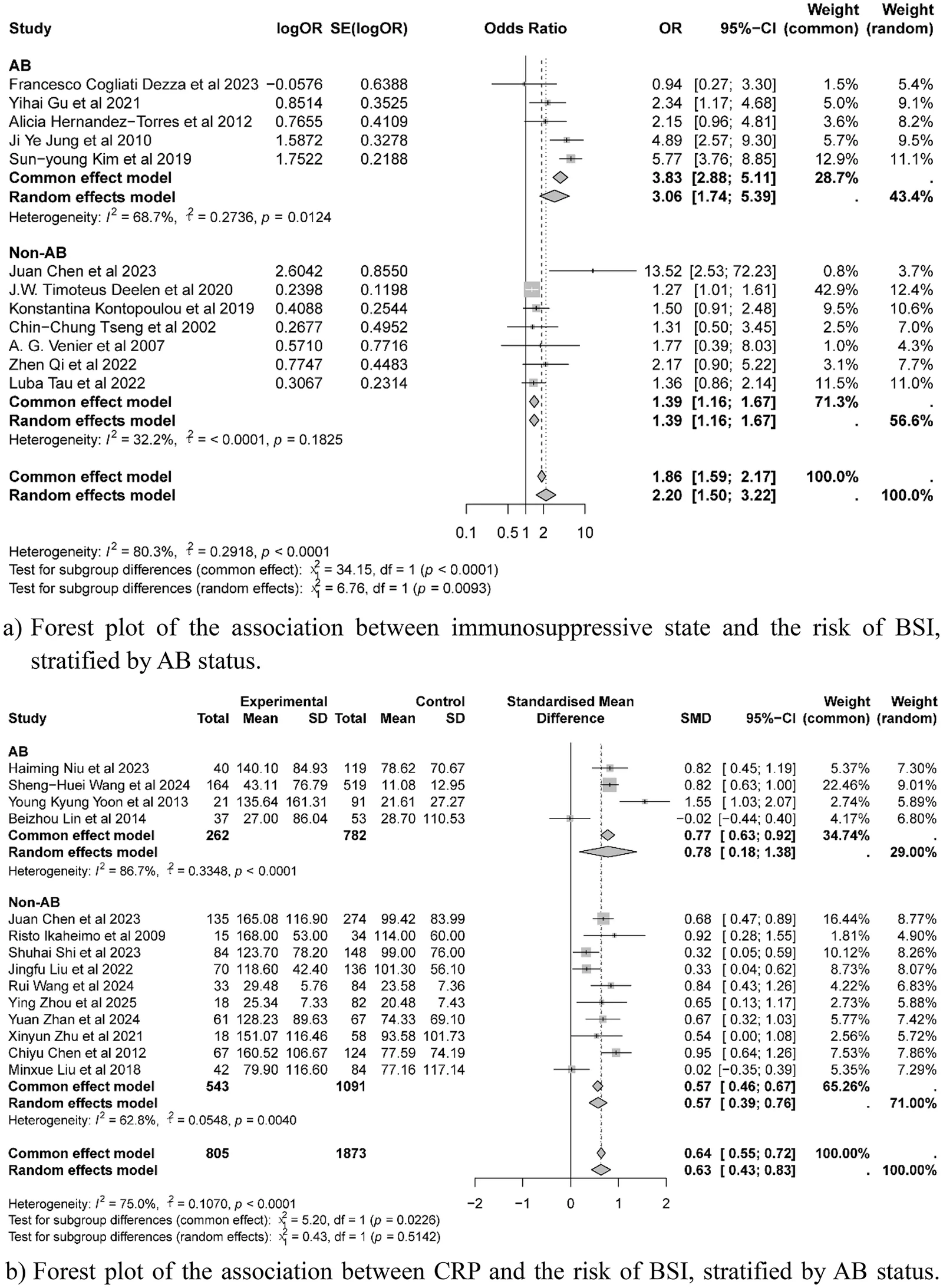

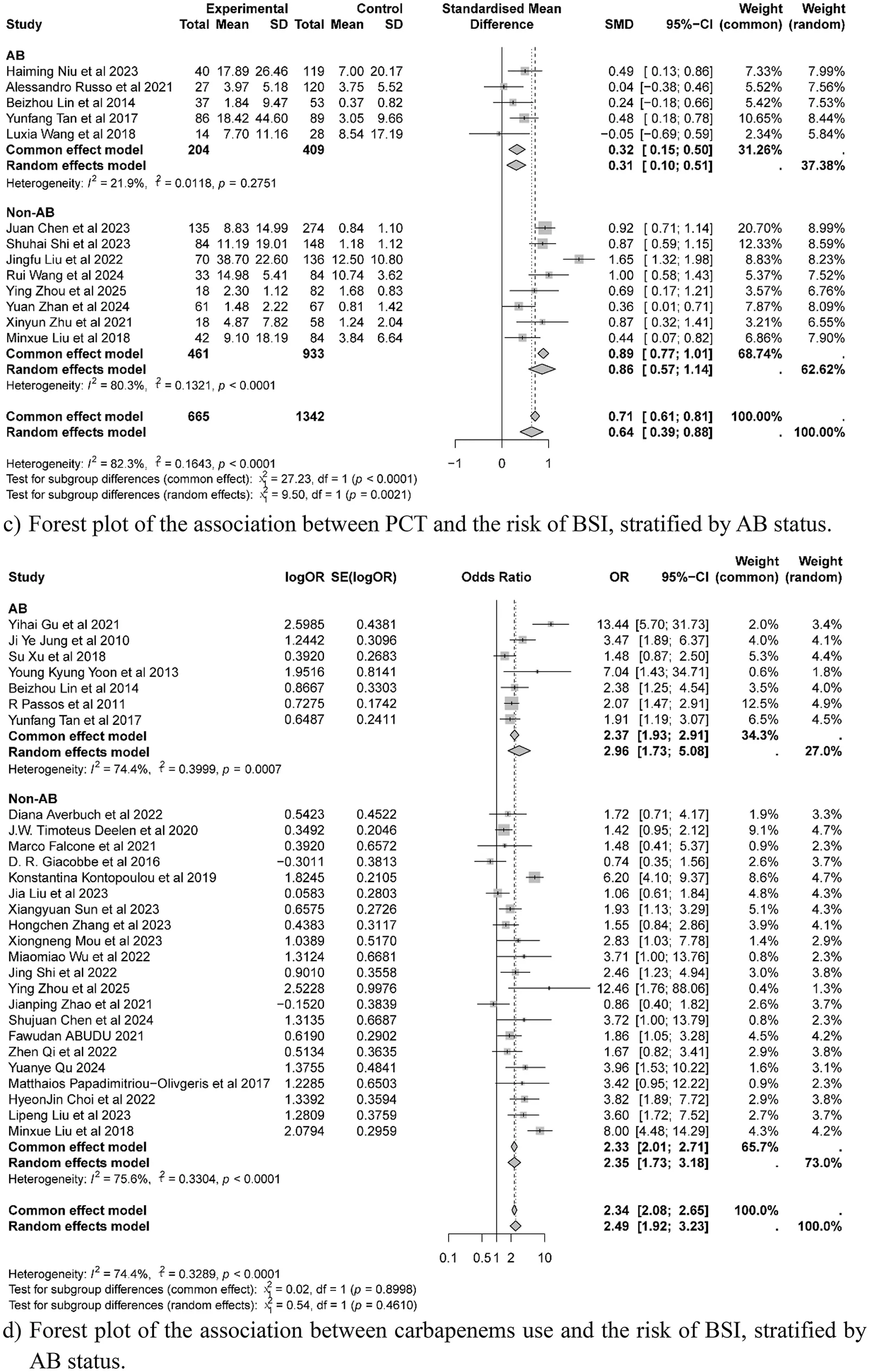

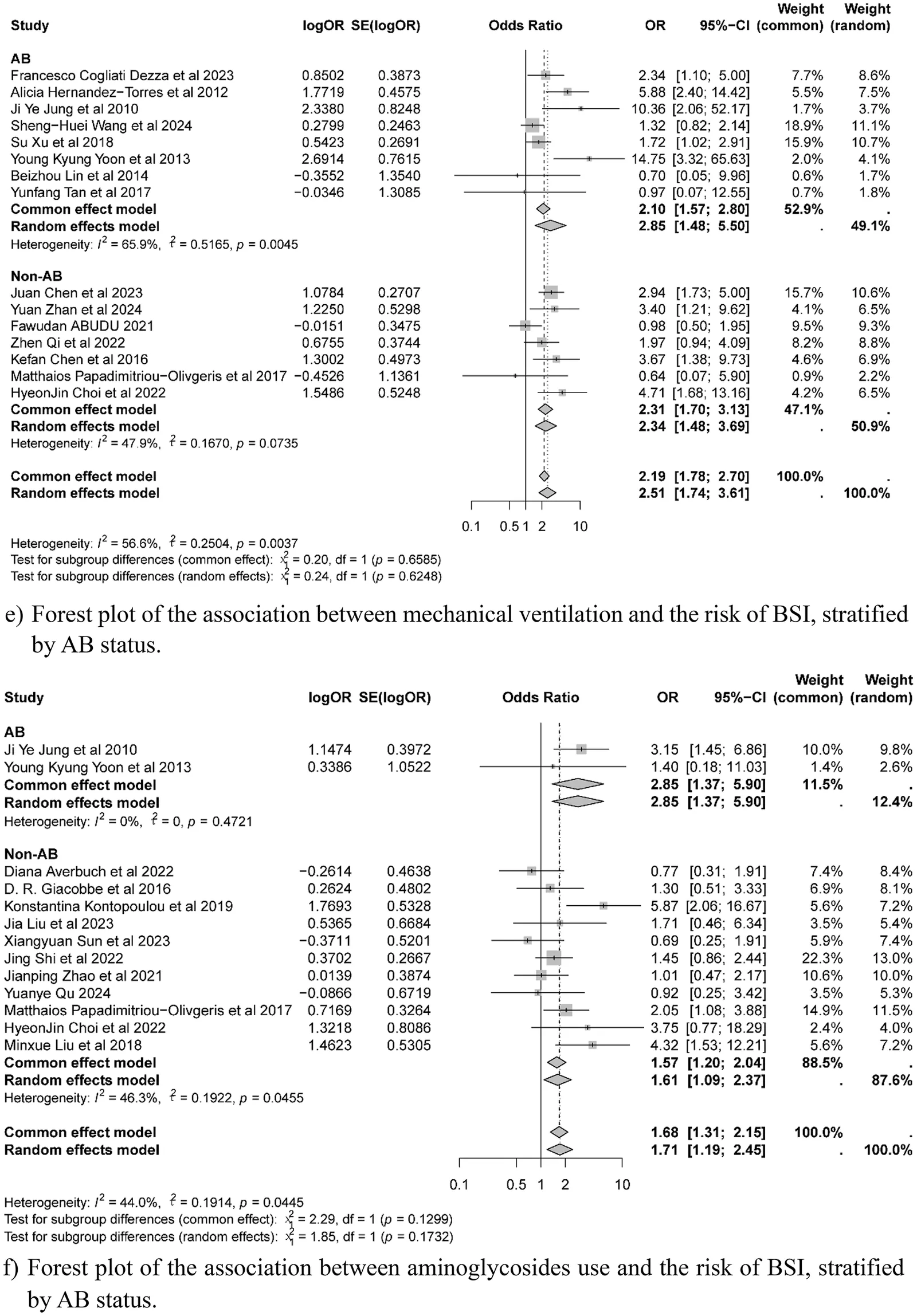

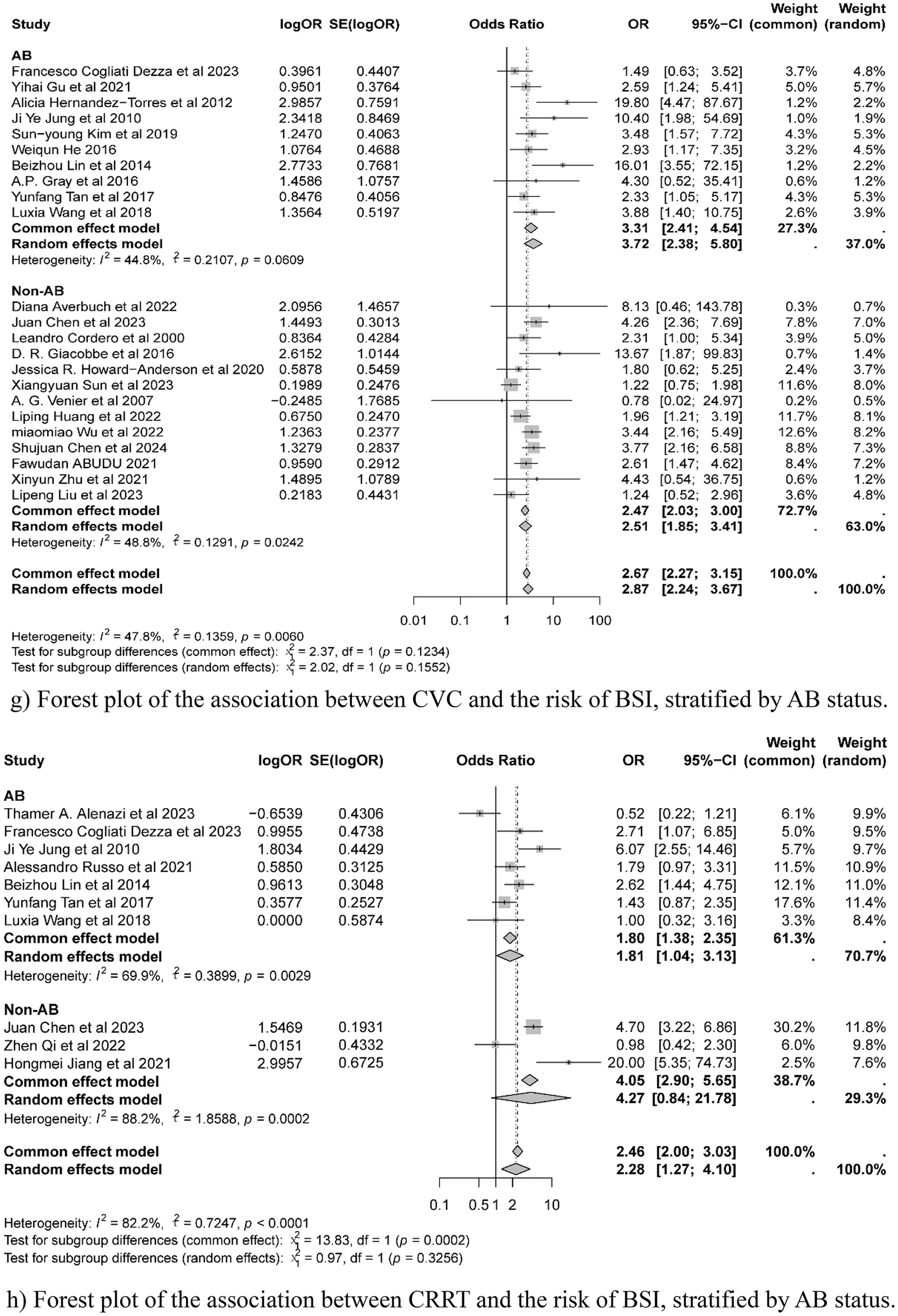
Forest plot of subgroup meta-analysis of risk factors for AB and Non-AB BSI

Among the 17 factors identified as significantly associated with BSI in the overall analysis, notable differences were observed between the AB and non-AB subgroups. Seven factors remained consistently significant in both subgroups: immunosuppressive state, PCT, CRP, carbapenem use, MV, aminoglycoside use, and the presence of a CVC. CRRT emerged as the only factor that was significantly associated with BSI exclusively in the AB subgroup (Fig. 2). All evaluated risk factors were further analyzed within the AB and non-AB subgroups. Detailed results are presented in Supplementary Table 2.

## Discussion

### Study strengths and comparison with previous literature

This study constitutes the first comprehensive meta-analysis to systematically evaluate risk factors for GNB-BSIs across a general inpatient population, with detailed stratification by resistance profile, including CR-GNB, and by specific pathogens, particularly AB. In contrast to earlier meta-analyses that primarily examined mortality outcomes [88, 89], focused on narrowly defined populations such as patients with COVID-19 or those admitted to ICUs [90, 91], or were limited to single pathogens such as *E. coli* [90], the present analysis provides a broad and integrated assessment of GNB-BSI risk factors across an unrestricted hospital population. This approach bridges the methodological gap between ICU-centric investigations and studies addressing the wider inpatient setting. Importantly, the overall GNB analysis in this study was not intended to homogenize distinct pathogens into a single clinical entity; rather, it was designed to identify broadly shared risk signals, while the resistance-stratified and pathogen-specific analyses were used to preserve clinically meaningful heterogeneity. This hierarchical framework allowed us to distinguish common predictors from those more specifically related to antimicrobial resistance or to particular organisms such as AB. Importantly, the strict stratification of susceptible versus resistant strains enabled differentiation between general infection-related risk factors and those specifically associated with antimicrobial resistance, thereby allowing the identification of determinants uniquely linked to resistant phenotypes rather than to BSIs. However, the pooled estimates for some variables should be interpreted cautiously, as substantial heterogeneity across the predominantly retrospective studies likely reflects differences in patient populations, clinical settings, and operational definitions, making these results better understood as summary association signals rather than precise, universally applicable effect sizes.

### Broadly shared risk factors and validation of guidelines

Several variables, including prior carbapenem use, MV, CVC use, immunosuppressive state, prior aminoglycoside exposure, PCT, and CRP, showed consistent statistical significance across the overall population and all predefined subgroups. Importantly, PCT and CRP should be interpreted as host-response biomarkers and diagnostic predictors rather than antecedent risk factors. Although current ESCMID guidelines [91] primarily recommend PCT for the diagnosis of sepsis, the present findings extend its clinical relevance by supporting its role in early risk stratification for GNB-BSI, thereby reinforcing its utility as a precise biomarker to guide timely antimicrobial initiation [92]. Prior carbapenem exposure was identified as a particularly strong predictor, especially for infections caused by resistant organisms such as CRAB. While this association may partly reflect confounding by indication, the biological plausibility of carbapenem-driven selective pressure on the gut and hospital microbiome is well established. A similar mechanism likely underlies the independent association observed with prior aminoglycoside use, which may facilitate the selection, colonization, or emergence of resistant Gram-negative pathogens. In this context, a history of carbapenem use should be interpreted as a dual indicator reflecting both patient vulnerability and the likelihood of resistant colonization. These findings provide strong empirical support for the 2024 IDSA guidance [93], which recommends broader empirical antimicrobial coverage in patients with such exposure histories [94, 95]. Immunosuppressive state also emerged as a significant broadly shared risk factor, underscoring the central role of host immune competence in preventing bacterial translocation and bloodstream invasion. This observation reinforces the need for tailored risk assessment and appropriately adjusted empirical therapy in immunocompromised patients, who exhibit heightened susceptibility to GNB-BSI. Furthermore, the identification of MV and CVC use as independent contributors aligns with existing CDC and SHEA/IDSA prevention recommendations [96], emphasizing that strict adherence to device maintenance bundles and timely removal remain essential strategies for minimizing infection risk associated with barrier disruption and biofilm formation [97].

### Pathogen-specific and resistance-associated drivers

Beyond the broadly shared predictors, distinct risk profiles were observed within specific resistance and pathogen subgroups. In patients with CR-GNB infections, hypoalbuminemia and IUC use emerged as significant predictors. Hypoalbuminemia reflects a combination of poor nutritional status and immune dysfunction, all of which may facilitate bacterial invasion and dissemination [98]. IUCs represent a well-recognized portal of entry for biofilm-forming organisms, including CRE [99]. Persistently elevated CRP levels in this subgroup may further reflect an exaggerated inflammatory response driven by treatment failure associated with resistance [100]. In the AB subgroup, CRRT was identified as a unique risk factor. CRRT likely functions as a convergence point for multiple risks, combining the need for prolonged invasive vascular access with the potential for microbial adherence and biofilm development on extracorporeal circuits [101]. This finding also supports the view that AB-related bloodstream infection has a more distinct environmental and device-associated risk profile, which may not be fully captured by broader pooled analyses of GNB-BSI. The stronger signal of host-related factors in the CRE subgroup may reflect the central roles of gastrointestinal colonization, antimicrobial selective pressure, and impaired nutritional or mucosal barrier status in CRE pathogenesis. By contrast, AB-related bloodstream infections may be influenced more by ICU exposure, environmental persistence, and device-associated transmission, potentially attenuating the relative contribution of host nutritional or hematologic markers. The smaller AB evidence base may also have limited statistical power to detect comparable associations. CKD may plausibly increase susceptibility to GNB-BSI through immune dysfunction, repeated healthcare exposure, vascular access, and frequent antimicrobial use [102–104], whereas the association with male sex should be interpreted cautiously given the potential influence of comorbidity burden, illness severity, device exposure, and residual confounding [105–107].

### Clinical implications

The multifactorial nature of the identified clinical predictors and associated factors highlights the need for structured risk stratification rather than a purely intuition-based approach. Although the present study does not provide a validated clinical prediction rule, it supports a risk-informed framework for identifying and prioritizing high-risk patients. Patients with CRAB infections, in particular, appear to experience a “double-hit” phenomenon, whereby risks associated with both the specific pathogen and its resistance profile converge. In this context, the more novel findings of this study lie in the pathogen-specific and resistance-associated niches, with CRRT representing a distinctive risk factor for AB-BSI, whereas hypoalbuminemia is uniquely associated with CR-GNB-BSI. These subgroup-specific signals may be more clinically informative than broadly recognized device-related risks alone when refining local surveillance and early management strategies. Preventive strategies in high-risk populations should extend beyond infection control alone and incorporate supportive measures such as nutritional optimization to address hypoalbuminemia, alongside rigorous daily evaluation of catheter necessity. From an AMS perspective, the dual role of carbapenems as both a life-saving therapy and resistance drivers necessitates careful, patient-specific empirical prescribing rather than uniform escalation. More specifically, the presence of high-risk features such as CRRT, hypoalbuminemia, prior carbapenem exposure, or immunosuppressive state should increase clinical suspicion for resistant Gram-negative pathogens and may justify earlier consideration of broader or more targeted empiric coverage, particularly in severely ill patients. However, any escalation strategy should be guided by local resistance epidemiology, institutional antibiograms, prior colonization history, and the patient’s overall clinical severity, rather than by isolated risk factors alone. Finally, increasing the frequency of PCT and CRP monitoring in high-risk patients may facilitate earlier detection of evolving infection, enabling timely intervention before overt clinical deterioration occurs.

### Limitations and future directions

Despite its comprehensive scope, this study has several limitations. First, the included studies varied in geographic setting, clinical practice, and operational definitions, including differences in exposure windows for prior antibiotic use, which may have influenced the pooled estimates. In particular, the timing and definition of aminoglycoside exposure were not uniform across studies, which may have introduced additional clinical heterogeneity. Second, because the study population included not only infected patients but also those with exposure to or colonization by the target organisms, the findings may be more informative for risk stratification and identification of high-risk patients than for direct empiric treatment decisions. Third, the included literature was geographically concentrated, with most studies originating from Asia, particularly China, and given the marked regional variation in antimicrobial resistance epidemiology, the generalizability of the findings to other settings may therefore be limited. In addition, substantial heterogeneity was observed for some variables, likely reflecting differences in patient populations, clinical settings, and study definitions across the predominantly retrospective observational literature. Accordingly, these pooled estimates should be interpreted as summary association signals rather than precise and universally applicable effect sizes. Finally, because this meta-analysis was based on observational studies, residual confounding, publication bias, and limited power in some subgroup analyses cannot be excluded. These findings should also be interpreted cautiously because relatively few included studies reported standardized multivariable-adjusted effect estimates; further studies with more consistent multivariable reporting are needed to better confirm the independence of the identified risk factors. Future studies should further clarify the molecular and epidemiological mechanisms underlying these findings, improve geographic representation, and develop and validate clinically applicable risk prediction models. In addition, well-designed prospective interventional studies are needed to determine whether targeted strategies such as nutritional optimization and enhanced catheter management can effectively reduce the incidence of GNB-BSI, particularly those caused by resistant organisms.

## Conclusions

To our knowledge, this study represents the first meta-analysis to comprehensively synthesize risk factors for GNB-BSI across distinct pathogen- and resistance-specific phenotypes. PCT, prior carbapenem use, MV, CVC use, immunosuppressive state, CRP, and prior aminoglycoside exposure were identified as dominant broadly shared predictors. Clear subgroup-specific patterns were also observed, with hypoalbuminemia and IUC use characterizing CR-GNB infections and CRRT emerging as a distinguishing factor for AB infections. Collectively, these findings highlight the urgent need to optimize carbapenem stewardship and reinforce infection prevention measures related to invasive devices. Patients should be stratified according to the cumulative burden of identified risk factors, with those exhibiting high-risk profiles regarded as priority candidates for enhanced surveillance and tailored empirical therapy, particularly in the context of CRAB. Future efforts should focus on validating these results through predictive modeling and prospective interventional studies.

## Supplementary Information

Below is the link to the electronic supplementary material.

Supplementary Material 1

Supplementary Material 2

## Data Availability

All the data in this review are included in the manuscript.

## Abbreviations

BSI: Bloodstream infection
GNB: Gram-negative bacteria
AB: *Acinetobacter baumannii*
Ent.: Enterobacter species
KP: Klebsiella pneumoniae
PA: Pseudomonas aeruginosa
CR-GNB: Carbapenem-resistant Gram-negative bacteria
CRAB: Carbapenem-resistant Acinetobacter baumannii
GNB-BSI: Gram-negative bacteria bloodstream infection
CNKI: China National Knowledge Infrastructure
OR: Odds ratio
CI: Confidence interval
SD: Standard deviation
NOS: Newcastle-Ottawa Scale
AHRQ: Agency for Healthcare Research and Quality
SMD: Standardized mean difference
CCI: Charlson Comorbidity Index
COPD: Chronic obstructive pulmonary disease
CKD: Chronic kidney disease
CRP: C-reactive protein
WBC: White blood cell
PCT: Procalcitonin
ALB: Albumin
PLT: Platelet
MV: Mechanical ventilation
CRRT: Continuous renal replacement therapy
CVC: Central venous catheter
IUC: Indwelling urinary catheter
CR: Carbapenem-resistant
Non-CR: Non-carbapenem-resistant
