# Supplementary material for: Risk factors for Gram-negative bacterial bloodstream infections: a systematic review and meta-analysis with subgroup analyses for carbapenem-resistant organisms and Acinetobacter baumannii
Source: BMC Infect Dis. 2026 Aug 8;26:1952. doi: 10.1186/s12879-026-14108-6 (PMC13737500; doi:10.1186/s12879-026-14108-6)
Supplement: Supplementary file 1 [file 12879_2026_14108_MOESM1_ESM.docx]

**Appendix A. Supplementary data**

**Supplementary Table 1** **Subgroup meta-analysis of risk factors for CRAB BSI**

| **Risk factors** | **CRAB** | | |
| --- | --- | --- | --- |
|  | Number of  studies | Pooled OR (95%CI) / SMD [95%CI] | *P value* |
| *Age | 3 | -0.20 [-0.35; -0.06] | 0.006 |
| Gender, male | 4 | 1.20 (0.90; 1.59) | 0.212 |
| *CCI | -- | -- | -- |
| COPD | 2 | 1.05 (0.54; 2.01) | 0.894 |
| Heart failure | 4 | 1.02 (0.72; 1.44) | 0.921 |
| Immunosuppressive state | 2 | 1.69 (0.86; 3.33) | 0.129 |
| Diabetes mellitus | 4 | 0.98 (0.58; 1.66) | 0.940 |
| Hypertension | 3 | 0.94 (0.41; 2.15) | 0.885 |
| CKD | 3 | 2.44 (0.63; 9.49) | 0.200 |
| Cancer | 5 | 1.18 (0.56; 2.49) | 0.654 |
| Cancer-Solid | 2 | 1.04 (0.29; 3.72) | 0.950 |
| Cancer-Non-solid | 2 | 2.58 (1.06; 6.29) | 0.037 |
| *CRP | 2 | 0.82 [0.65; 0.98] | <0.001 |
| *WBC | -- | -- | -- |
| *PCT | -- | -- | -- |
| *Neutrophil | -- | -- | -- |
| *ALB | 2 | -0.12 [-0.28; 0.03] | 0.123 |
| *PLT | -- | -- | -- |
| Carbapenems use | -- | -- | -- |
| MV | 3 | 2.46 (1.06; 5.72) | 0.036 |
| *MV days | -- | -- | -- |
| Immunosuppression therapy | -- | -- | -- |
| Aminoglycosides use | -- | -- | -- |
| CRRT | -- | -- | -- |
| Surgery | 2 | 3.64 (0.19; 70.40) | 0.393 |
| CVC | 3 | 4.63 (0.91; 23.44) | 0.064 |
| IUC | -- | -- | -- |
| Cephalosporins use/exposure | -- | -- | -- |
| Fluoroquinolones use | -- | -- | -- |

Note: *Indicates a continuous variable. Results are presented as SMD (95% CI).

**Supplementary Table 2 Subgroup meta-analysis of risk factors for AB and Non-AB BSI**

| **Risk factors** | **AB** | | | **Non-AB** | | |
| --- | --- | --- | --- | --- | --- | --- |
|  | Number of  studies | Pooled OR (95%CI) / SMD [95%CI] | *P value* | Number of  studies | Pooled OR (95%CI) / SMD [95%CI] | *P value* |
| *Age | 12 | -0.05 [-0.13; 0.04] | 0.286 | 35 | 0.07 [-0.06; 0.21] | 0.293 |
| Gender, male | 15 | 1.16 (0.98; 1.38) | 0.083 | 47 | 1.14 (1.05; 1.24) | 0.002 |
| *CCI | 3 | 0.07 [-0.17; 0.30] | 0.572 | 7 | 0.06 [-0.17; 0.28] | 0.616 |
| COPD | 8 | 1.05 (0.64; 1.73) | 0.847 | 9 | 1.01 (0.58; 1.76) | 0.967 |
| Heart failure | 7 | 0.94 (0.69; 1.29) | 0.708 | 5 | 0.89 (0.55; 1.43) | 0.621 |
| Immunosuppressive state | 5 | 3.06 (1.74; 5.39) | <0.001 | 7 | 1.39 (1.16; 1.67) | <0.001 |
| Diabetes mellitus | 12 | 1.01 (0.83; 1.23) | 0.892 | 33 | 1.13 (1.00; 1.28) | 0.043 |
| Hypertension | 7 | 0.91 (0.70; 1.17) | 0.455 | 16 | 0.93 (0.79; 1.09) | 0.394 |
| Chronic kidney disease | 7 | 1.60 (0.97; 2.64) | 0.068 | 13 | 1.24 (0.87; 1.76) | 0.243 |
| Cancer | 13 | 1.14 (0.65; 1.97) | 0.652 | 31 | 1.52 (1.31; 1.77) | <0.001 |
| Cancer-Solid | 4 | 1.25 (0.54; 2.87) | 0.606 | 11 | 0.69 (0.54; 0.88) | 0.003 |
| Cancer-Non-solid | 4 | 2.45 (0.64; 9.42) | 0.192 | 10 | 1.87 (1.20; 2.92) | 0.006 |
| *CRP | 4 | 0.78 [0.18; 1.38] | 0.010 | 10 | 0.57 [0.39; 0.76] | <0.001 |
| *WBC | 3 | -0.17 [-0.39; 0.04] | 0.117 | 12 | 0.35 [0.07; 0.63] | 0.015 |
| *PCT | 5 | 0.32 [0.15; 0.50] | <0.001 | 8 | 0.86 [0.57; 1.14] | <0.001 |
| *Neutrophil | -- | -- | -- | 7 | 0.23 [-0.01; 0.47] | 0.056 |
| *ALB | 2 | -0.12 [-0.28; 0.03] | 0.123 | 6 | -0.32 [-0.46; -0.18] | <0.001 |
| *PLT | 3 | -0.07 [-0.30; 0.15] | 0.514 | 5 | -0.38 [-0.76; -0.00] | 0.048 |
| Carbapenems use | 7 | 2.96 (1.73; 5.08) | <0.001 | 21 | 2.35 (1.73; 3.18) | <0.001 |
| Mechanical ventilation | 8 | 2.85 (1.48; 5.50) | 0.002 | 7 | 2.31 (1.70; 3.13) | <0.001 |
| *Mechanical ventilation days | -- | -- | -- | 4 | -0.04 [-0.63; 0.56] | 0.906 |
| Immunosuppression therapy | -- | -- | -- | 13 | 1.72 (1.21; 2.44) | 0.002 |
| Aminoglycosides use | 2 | 2.85 (1.37; 5.90) | 0.005 | 11 | 1.57 (1.20; 2.04) | 0.001 |
| CRRT | 7 | 1.81 (1.04; 3.13) | 0.035 | 3 | 4.27 (0.84; 21.78) | 0.080 |
| Surgery | 8 | 1.92 (0.84; 4.39) | 0.12 | 17 | 1.20 (0.89; 1.62) | 0.237 |
| Central venous catheter | 10 | 3.31 (2.41; 4.54) | <0.001 | 13 | 2.47 (2.03; 3.00) | <0.001 |
| Indwelling urinary catheter | 6 | 1.53 (0.75; 3.15) | 0.246 | 17 | 1.51 (1.03; 2.23) | 0.035 |
| Cephalosporins use/exposure | 4 | 2.03 (0.67; 6.19) | 0.211 | 14 | 1.10 (0.74; 1.63) | 0.652 |
| Fluoroquinolones use | 4 | 1.87 (0.79; 4.39) | 0.152 | 16 | 1.29 (0.88; 1.89) | 0.188 |

Note: *Indicates a continuous variable. Results are presented as SMD (95% CI).

**Supplementary Table 3. Newcastle-Ottawa Scale quality assessment of cohort studies**

| First author and year | **SELECTION** | | | | **COMPARABILITY** | **OUTCOME** | | | **Total Score** | **Grade** |
| --- | --- | --- | --- | --- | --- | --- | --- | --- | --- | --- |
|  | 1) Representativeness of the exposed cohort | 2) Selection of the non-exposed cohort | 3) Ascertainment of exposure | 4) Demonstration that outcome of interest was not present at start of study | 1) Comparability of cohorts on the basis of the design or analysis | 1) Assessment of outcome | 2) Was follow-up long enough for outcomes to occur | 3) Adequacy of follow up of cohorts |  |  |
| Thamer A. Alenazi et al | 1 | 1 | 1 | 1 | 2 | 1 | 1 | 1 | 9 | high grade |
| Anucha Apisarnthanarak et al | 1 | 1 | 0 | 1 | 1 | 1 | 1 | 1 | 7 | high grade |
| Diana Averbuch et al | 1 | 1 | 1 | 1 | 1 | 1 | 1 | 1 | 8 | high grade |
| Evgeni Brotfain et al | 1 | 1 | 1 | 1 | 2 | 1 | 1 | 1 | 9 | high grade |
| Juan Chen et al | 1 | 1 | 1 | 1 | 1 | 1 | 1 | 1 | 8 | high grade |
| Francesco Cogliati Dezza et al | 1 | 1 | 1 | 1 | 2 | 1 | 1 | 1 | 9 | high grade |
| Leandro Cordero et al | 0 | 1 | 1 | 1 | 0 | 1 | 1 | 1 | 6 | moderate grade |
| Leandro Cordero et al | 0 | 1 | 1 | 1 | 0 | 1 | 1 | 1 | 6 | moderate grade |
| Patricia Cornejo-Juárez et al | 0 | 1 | 1 | 1 | 1 | 1 | 1 | 1 | 7 | high grade |
| J.W. Timotëus Deelen et al | 1 | 1 | 1 | 1 | 0 | 1 | 1 | 1 | 7 | high grade |
| Marco Falcone et al | 1 | 1 | 1 | 1 | 1 | 1 | 1 | 1 | 8 | high grade |
| D. R. Giacobbe et al | 1 | 1 | 1 | 1 | 2 | 1 | 1 | 1 | 9 | high grade |
| Alicia Herna´ndez-Torres et al | 1 | 1 | 1 | 1 | 1 | 1 | 1 | 1 | 8 | high grade |
| Jessica R. Howard-Anderson et al | 1 | 1 | 1 | 1 | 2 | 1 | 1 | 1 | 9 | high grade |
| Risto Ikäheimo et al | 0 | 1 | 1 | 1 | 0 | 1 | 1 | 1 | 6 | moderate grade |
| Ji Ye Jung et al | 1 | 1 | 1 | 1 | 2 | 1 | 1 | 1 | 9 | high grade |
| Sung-Woon Kang et al | 1 | 1 | 1 | 1 | 2 | 1 | 1 | 1 | 9 | high grade |
| Sun-young Kim et al | 1 | 1 | 1 | 1 | 2 | 1 | 1 | 1 | 9 | high grade |
| B. J. Liss et al | 1 | 1 | 1 | 1 | 2 | 1 | 1 | 1 | 9 | high grade |
| Jia Liu et al | 0 | 1 | 1 | 1 | 2 | 1 | 1 | 1 | 8 | high grade |
| Jonas Marschall et al | 1 | 1 | 1 | 1 | 1 | 1 | 1 | 1 | 8 | high grade |
| Elizabeth Temkin et al | 1 | 1 | 1 | 1 | 2 | 1 | 1 | 1 | 9 | high grade |
| Chin-Chung Tseng et al | 0 | 1 | 1 | 1 | 2 | 1 | 1 | 1 | 8 | high grade |
| Young Kyung Yoon et al | 1 | 1 | 1 | 1 | 2 | 1 | 1 | 1 | 9 | high grade |
| Shuhai Shi et al | 0 | 1 | 1 | 1 | 2 | 1 | 1 | 0 | 7 | high grade |
| Chunhui Xu et al | 0 | 1 | 1 | 1 | 2 | 1 | 1 | 1 | 8 | high grade |
| Han Meng et al | 1 | 1 | 1 | 1 | 2 | 1 | 1 | 1 | 9 | high grade |
| Yalu Ren et al | 0 | 1 | 1 | 1 | 0 | 1 | 1 | 1 | 6 | moderate grade |
| Shujuan Chen et al | 1 | 1 | 1 | 1 | 2 | 1 | 1 | 1 | 9 | high grade |
| Guoyao Ding | 1 | 1 | 1 | 1 | 2 | 1 | 1 | 1 | 9 | high grade |
| Yuanye Qu | 0 | 1 | 1 | 1 | 2 | 1 | 1 | 1 | 8 | high grade |
| Hongmei Jiang et al | 0 | 1 | 1 | 1 | 1 | 1 | 1 | 1 | 7 | high grade |
| Xinyun Zhu et al | 0 | 1 | 1 | 1 | 2 | 1 | 1 | 1 | 8 | high grade |
| Matthaios Papadimitriou-Olivgeris et al | 0 | 1 | 1 | 1 | 2 | 1 | 1 | 1 | 8 | high grade |
| HyeonJin Choi et al | 1 | 1 | 1 | 1 | 0 | 1 | 1 | 1 | 7 | high grade |
| V. Galfo et al | 1 | 1 | 1 | 1 | 1 | 1 | 1 | 1 | 8 | high grade |
| R Passos et al | 1 | 1 | 1 | 1 | 1 | 1 | 1 | 1 | 8 | high grade |
| Yunfang Tan et al | 1 | 1 | 1 | 1 | 1 | 1 | 1 | 1 | 8 | high grade |
| Lipeng Liu et al | 0 | 1 | 1 | 1 | 2 | 1 | 1 | 1 | 8 | high grade |
| Chenfeng Zhao et al | 0 | 1 | 1 | 1 | 0 | 1 | 1 | 1 | 6 | moderate grade |

**Supplementary Table 4. Newcastle-Ottawa Scale quality assessment of case-control studies**

| First author and year | **SELECTION** | | | | **COMPARABILITY** | **EXPOSURE** | | | **Total Score** | **Grade** |
| --- | --- | --- | --- | --- | --- | --- | --- | --- | --- | --- |
|  | 1) Is the Case Definition Adequate? | 2) Representativeness of the Cases | 3) Selection of Controls | 4) Definition of Controls | 1) Comparability of Cases and Controls on the Basis of the Design or Analysis | 1) Ascertainment of exposure | 2) Same method of ascertainment for cases and controls | 3) Non-Response rate |  |  |
| Yihai Gu et al | 1 | 1 | 0 | 1 | 2 | 1 | 1 | 0 | 7 | high grade |
| Konstantina Kontopoulou et al | 1 | 1 | 0 | 1 | 1 | 1 | 1 | 0 | 6 | moderate grade |
| Haiming Niu et al | 1 | 1 | 0 | 1 | 2 | 1 | 1 | 0 | 7 | high grade |
| Seon Jung Oh et al | 1 | 0 | 0 | 1 | 0 | 1 | 1 | 0 | 4 | moderate grade |
| Emmanuel Roilides et al | 1 | 0 | 1 | 1 | 1 | 1 | 1 | 0 | 6 | moderate grade |
| Xiangyuan Sun et al | 1 | 1 | 1 | 1 | 1 | 1 | 1 | 0 | 7 | high grade |
| Willize E. van der Starre et al | 1 | 0 | 1 | 1 | 0 | 1 | 1 | 1 | 6 | moderate grade |
| A. G. Venier et al | 1 | 0 | 1 | 1 | 1 | 1 | 1 | 0 | 6 | moderate grade |
| Sheng-Huei Wang et al | 1 | 1 | 1 | 1 | 2 | 1 | 1 | 0 | 8 | high grade |
| Su Xu et al | 1 | 1 | 1 | 1 | 2 | 1 | 1 | 0 | 8 | high grade |
| Hongchen Zhang et al | 1 | 0 | 1 | 1 | 2 | 1 | 1 | 1 | 8 | high grade |
| Li Yan et al | 1 | 1 | 1 | 1 | 2 | 1 | 1 | 1 | 9 | high grade |
| Liping Huang et al | 1 | 1 | 1 | 1 | 2 | 1 | 1 | 0 | 8 | high grade |
| Xiongneng Mou et al | 1 | 1 | 1 | 1 | 2 | 1 | 1 | 0 | 8 | high grade |
| Jingfu Liu et al | 1 | 1 | 1 | 1 | 1 | 1 | 1 | 0 | 7 | high grade |
| Rui Wang et al | 1 | 0 | 1 | 1 | 2 | 1 | 1 | 0 | 7 | high grade |
| Chunhua Li et al | 1 | 1 | 1 | 1 | 1 | 1 | 1 | 0 | 7 | high grade |
| Jing Shi et al | 1 | 1 | 1 | 1 | 1 | 1 | 1 | 0 | 7 | high grade |
| Weiqun He | 1 | 1 | 1 | 1 | 2 | 1 | 1 | 0 | 8 | high grade |
| Ying Zhou et al | 1 | 0 | 1 | 1 | 1 | 1 | 1 | 0 | 6 | moderate grade |
| Jianping Zhao et al | 1 | 1 | 1 | 1 | 0 | 1 | 1 | 0 | 6 | moderate grade |
| Yuan Zhan et al | 1 | 1 | 1 | 1 | 1 | 1 | 1 | 0 | 7 | high grade |
| Haitao Ren et al | 1 | 1 | 1 | 1 | 2 | 1 | 1 | 0 | 8 | high grade |
| Fawudan ABUDU | 1 | 0 | 1 | 1 | 2 | 1 | 1 | 0 | 7 | high grade |
| Jihong Li et al | 1 | 1 | 1 | 1 | 0 | 1 | 1 | 0 | 6 | moderate grade |
| Beizhou Lin et al | 1 | 1 | 1 | 1 | 2 | 1 | 1 | 0 | 8 | high grade |
| Jiaping Lin | 1 | 1 | 1 | 1 | 2 | 1 | 1 | 0 | 8 | high grade |
| Zhen Qi et al | 1 | 0 | 1 | 1 | 1 | 1 | 1 | 0 | 6 | moderate grade |
| Kefan Chen et al | 1 | 1 | 1 | 1 | 1 | 1 | 1 | 0 | 7 | high grade |
| Chiyu Chen et al | 1 | 1 | 1 | 1 | 2 | 1 | 1 | 0 | 8 | high grade |
| Luba Tau et al | 1 | 1 | 1 | 1 | 2 | 1 | 1 | 0 | 8 | high grade |
| A.P. Gray et al | 1 | 1 | 1 | 1 | 1 | 1 | 1 | 1 | 8 | high grade |
| Minxue Liu et al | 1 | 0 | 1 | 1 | 2 | 1 | 1 | 0 | 7 | high grade |
| Luxia Wang et al | 1 | 1 | 1 | 1 | 1 | 1 | 1 | 0 | 7 | high grade |

**Supplementary Table 5. Agency for Healthcare Research and Quality assessment of cross-sectional studies**

| First author and year | 1. Define the source of information (survey, record review). | 2. List inclusion and exclusion criteria for exposed and unexposed subjects (cases and controls) or refer to previous publications. | 3. Indicate time period used for identifying patients. | 4. Indicate whether or not subjects were consecutive if not population-based. | 5. Indicate if evaluators of subjective components of study were masked to other aspects of the participants. | 6. Describe any assessments undertaken for quality assurance purposes (e.g., test/retest of primary outcome measurements). | 7. Explain any patient exclusions from analysis. | 8. Describe how confounding was assessed and/or controlled. | 9. If applicable, explain how missing data were handled in the analysis. | 10. Summarize patient response rates and completeness of data collection. | 11. Clarify what follow-up, if any, was expected and the percentage of patients for which incomplete data or follow-up was obtained. | Total | Grade |
| --- | --- | --- | --- | --- | --- | --- | --- | --- | --- | --- | --- | --- | --- |
|  | 1) Yes 2) No 3) Unclear | 1) Yes 2) No 3) Unclear | 1) Yes 2) No 3) Unclear | 1) Yes 2) No 3) Unclear | 1) Yes 2) No 3) Unclear | 1) Yes 2) No 3) Unclear | 1) Yes 2) No 3) Unclear | 1) Yes 2) No 3) Unclear | 1) Yes 2) No 3) Unclear | 1) Yes 2) No 3) Unclear | 1) Yes 2) No 3) Unclear |  |  |
| Alessandro Russo et al | 1 | 1 | 1 | 1 | 0 | 1 | unclear | 1 | unclear | 1 | unclear | 7 | moderate grade |
| Miaomiao Wu et al | 1 | 1 | 1 | 1 | 0 | 1 | unclear | 1 | unclear | 1 | unclear | 7 | moderate grade |

**Supplementary Table 6** **Library search strings**

**A. Search Strategy for English Databases (PubMed, Embase, Web of Science, and the Cochrane Library)**

Conceptual Search Blocks:

#1 (Pathogen Concept 1: General Gram-Negative Bacteria)

("Gram Negative Bacteria"[Mesh]) OR "Gram Negative Bacteria" OR "GNB" OR "Carbapenem-resistant Organism" OR "CRO" OR "carbapenem resistance" OR "carbapenemase-producing" OR "CRGNB"

#2 (Pathogen Concept 2: Acinetobacter baumannii)

("Acinetobacter baumannii"[Mesh]) OR "Acinetobacter baumannii" OR "A.baumannii" OR "Acinetobacter" OR "CRAB"

#3 (Pathogen Concept 3: Enterobacteriaceae)

("Enterobacteriaceae"[Mesh]) OR "Enterobacteriaceae" OR ("Escherichia"[Mesh]) OR "Escherichia" OR "Escherichia coli" OR "E.coli" OR "carbapenem-resistant escherichia coli" OR "carbapenem-resistant Enterobacteriaceae" OR "CRE" OR "CREC"

#4 (Pathogen Concept 4: Klebsiella)

("Klebsiella"[Mesh]) OR "klebsiella pneumoniae" OR "K.pneumoniae" OR "Klebsiella" OR "carbapenem-resistant klebsiella pneumoniae" OR "CRKP"

#5 (Pathogen Concept 5: Pseudomonas aeruginosa)

("Pseudomonas aeruginosa"[Mesh]) OR "Pseudomonas aeruginosa" OR "P. aeruginosa" OR "Pseudomonas" OR "carbapenem-resistant pseudomonas aeruginosa" OR "CRPA"

#6 (Infection Type Concept)

("Sepsis"[Mesh]) OR ("Bacteremia"[Mesh]) OR "bacteraemia" OR "bloodstream infection" OR "BSI" OR "septicemia" OR "sepsis"

#7 (Risk Factor Concept)

("Risk Factors"[Mesh]) OR "association" OR "impact" OR "exposure" OR "risk factor" OR "predictor" OR "Susceptibility factor" OR "risk score" OR "correlates" OR "risk determinant" OR "risk indicator" OR "hazard factor" OR "contributing factor" OR "susceptibility" OR "vulnerable factor" OR "predisposing factor" OR "risk marker" OR "risk index"

#8 (Study Design Filter: Observational Studies)

("Observational Study"[pt] OR "Observational Studies as Topic"[Mesh] OR "Cohort Studies"[Mesh] OR "Case-Control Studies"[Mesh] OR "Cross-Sectional Studies"[Mesh] OR Epidemiologic Stud*[tiab] OR Observational Stud*[tiab] OR Cohort[tiab] OR "Follow-Up"[tiab] OR Longitudinal*[tiab] OR Prospectiv*[tiab] OR Retrospectiv*[tiab] OR "Case-Control"[tiab] OR "Cross-Sectional"[tiab]) NOT ("Animals"[Mesh] NOT ("Humans"[Mesh] AND "Animals"[Mesh]))

**Final Search Combination:**

**(#1 OR #2 OR #3 OR #4 OR #5) AND #6 AND #7 AND #8**

**B. Search Strategy for Chinese Databases (CNKI, Wanfang, VIP, and CBM)**

Conceptual Search Blocks (Translated Terms):

#1 (Pathogen):

("Gram-negative bacteria" OR "Acinetobacter baumannii" OR "Enterobacter" OR "Enterobacteriaceae" OR "Escherichia coli" OR "Klebsiella pneumoniae" OR "Pseudomonas aeruginosa" OR "AB" OR "KP" OR "PA" OR "CRO" OR "CRAB" OR "CRE" OR "CREC" OR "CRKP" OR "CRPA" OR "CRGNB" OR "GNB")

#2 (Infection Type):

("Bloodstream infection" OR "Sepsis" OR "Bacteremia" OR "Septicemia" OR "BSI" OR "Hematogenous infection")

#3 (Outcome: Risk Factors):

("Risk factor" OR "Influencing factor" OR "Predictor" OR "Susceptibility factor" OR "Risk indicator" OR "Associated factor")

**Final Search Combination:**

**#1 AND #2 AND #3**

**Supplementary Assessment of Publication Bias for Cancer**

Egge's test for cancer was statistically significant, suggesting possible funnel plot asymmetry and potential small-study effects. Visual inspection of the original funnel plot, however, did not reveal marked asymmetry. To further explore this issue, trim-and-fill analysis and leave-one-out sensitivity analysis were conducted.

The original pooled OR for cancer was 1.28 (95% CI 1.03–1.60). After trim-and-fill adjustment, the pooled OR increased to 1.82 (95% CI 1.42–2.34). This pattern suggests that the observed asymmetry does not support a simple interpretation of effect-size inflation due to publication bias. Instead, it may reflect more complex small-study effects or underlying between-study heterogeneity.

Leave-one-out sensitivity analysis showed that sequential omission of each individual study did not materially alter the pooled estimate, with the recalculated ORs remaining highly consistent across iterations. This finding suggests that the association was not driven by any single study and supports the overall robustness of the pooled result.

Overall, these findings suggest that the association between cancer and the outcome should be interpreted cautiously. However, the available exploratory analyses do not indicate that the observed result was driven solely by a single influential study or by a simple form of publication bias.

Supplementary Figure 1 Original funnel plot for the association between cancer and GNB-BSI

Supplementary Figure 2 Funnel plot after trim-and-fill adjustment for the association between cancer and GNB-BSI

Supplementary Figure 3 Forest plot after trim-and-fill adjustment for the association between cancer and GNB-BSI

Supplementary Figure 4 Leave-one-out sensitivity analysis for the association between cancer and GNB-BSI

**Supplementary sensitivity analysis based on multivariable-adjusted estimates**

To further assess the robustness of the main findings, supplementary meta-analyses restricted to multivariable-adjusted effect estimates were conducted for eligible variables with sufficient data availability. Owing to the limited number of studies, these analyses were presented as sensitivity analyses in the Supplementary Materials and were not further stratified by subgroup. Overall, the direction of association for these variables was generally consistent with that of the primary analysis, supporting the robustness of the main findings while still warranting cautious interpretation.

Notably, gender was not significantly associated with the outcome in the meta-analysis restricted to multivariable-adjusted estimates, in contrast to the primary analysis. This difference suggests that the association observed in the main analysis may have been influenced by residual confounding, since sex may be correlated with other clinical factors such as comorbidity burden, severity of illness, healthcare exposure, or invasive device use rather than acting as an independent predictor. The relatively small number of eligible studies and the heterogeneity of adjustment strategies across studies may also have contributed to this inconsistency.

**1）Carbapenems use**

Supplementary Figure 5 Forest plot of multivariable-adjusted estimates for carbapenem use

**2）Central venous catheter**

Supplementary Figure 6 Forest plot of multivariable-adjusted estimates for central venous catheter use

**3）Mechanical ventilation**

Supplementary Figure 7 Forest plot of multivariable-adjusted estimates for mechanical ventilation

**4）Indwelling urinary catheter**

Supplementary Figure 8 Forest plot of multivariable-adjusted estimates for indwelling urinary catheter use

**5）Surgery**

Supplementary Figure 9 Forest plot of multivariable-adjusted estimates for surgery

**6）Cancer**

Supplementary Figure 10 Forest plot of multivariable-adjusted estimates for cancer

**7）Diabetes mellitus**

Supplementary Figure 11 Forest plot of multivariable-adjusted estimates for diabetes mellitus

**8）Gender**

Supplementary Figure 12 Forest plot of multivariable-adjusted estimates for gender
